# Polarization‐Enabled Piezoelectric Tellurium–Selenium (Te*_x_* Se_1–_ *_x_*) Thin Films for Memory Switching and Artificial Synaptic Functions

**DOI:** 10.1002/advs.202524308

**Published:** 2026-05-27

**Authors:** Chia‐Chen Chung, Mayur Chaudhary, Chia‐Hung Lo, Po‐Chien Lai, You‐Jie Lin, Ruei‐Hong Cyu, Yu‐Ren Peng, Bing‐Ni Gu, Zhao‐Feng Lou, Quynh Thi Le, Yi‐Jen Yu, Yen‐Fu Lin, Min‐Hung Lee, Ying‐Hao Chu, Chang‐Hong Shen, Der‐Hsien Lien, Yu‐Lun Chueh

**Affiliations:** ^1^ Department of Materials Science and Engineering National Tsing Hua University Hsinchu Taiwan; ^2^ Ph. D. Program in Prospective Functional Materials Industry National Tsing Hua University Hsinchu Taiwan; ^3^ Graduate School of Advanced Technology National Taiwan University Taipei Taiwan; ^4^ Instrument Center National Tsing Hua University Hsinchu Taiwan; ^5^ Department of Physics National Chung Hsing University Taichung Taiwan; ^6^ College of Semiconductor Research National Tsing‐Hua University Hsinchu Taiwan; ^7^ Department of Electronics Engineering National Yang Ming Chiao Tung University Hsinchu Taiwan; ^8^ Department of Physics National Sun Yat‐Sen University Kaohsiung Taiwan; ^9^ Department of Materials Science and Engineering Korea University Seoul Republic of Korea

**Keywords:** memory devices, piezoelectricity, tellurium‐based compounds, Te_x_Se_1‐x_ films, two‐dimensional (2D) materials

## Abstract

Two‐dimensional materials with piezoelectricity and polarization‐enabled electromechanical responses provide a promising basis for multifunctional electronics, including memory devices and neuromorphic computing. In this work, we explore cryogenic physical vapor deposition (cryogenic PVD)–grown Te_x_Se_1‐x_ thin films, a tellurium‐based compound with a tunable bandgap and enhanced non‐centrosymmetry, and examine their polarization‐associated electromechanical characteristics. A 10 nm Te_0.9_Se_0.1_ film exhibits a clear switchable electromechanical response with a piezoelectric coefficient d_33_ of 33 pm/V, together with stable piezoresponse under ambient conditions. Introducing a Se ratio of 0.1 is found to enhance the polarization behavior and domain response while maintaining the crystalline quality of the Te_x_Se_1‐x_ films. Memory devices based on Te_0.9_Se_0.1_ show retention beyond 2000 s and remain switchable up to 1000 cycles, with an HRS/LRS ratio exceeding 10^2^ under ± 20 V program/erase pulses when read at a drain voltage of 1 V. In addition, synaptic behavior is demonstrated with 92% image recognition accuracy at low energy consumption, suggesting potential for neuromorphic applications. These results highlight the potential of Te_x_Se_1‐x_ films as a polarization‐enabled piezoelectric semiconductor system for future low‐power memory and computing applications.

## Graphical abstract

Here, we demonstrate and investigate polarization‐enabled electromechanical responses in cryogenic physical vapor deposition (cryogenic PVD)‐deposited Te_x_Se_1‐x_ thin films, a tellurium‐based compound with a tunable bandgap and enhanced non‐centrosymmetry. Specifically, a 10 nm Te_0.9_Se_0.1_ thin film exhibits a clear switchable electromechanical response with a piezoelectric coefficient d_33_ of 33 pm/V and a stable piezoresponse under ambient conditions. Se incorporation enhances polarization and domain behavior while preserving crystallinity. Te_0.9_Se_0.1_ memory devices achieve >2000 s retention, >1000‐cycle endurance, HRS/LRS > 10^2^, and 92% image recognition accuracy at low energy, highlighting potential for scalable, low‐power computing and memory systems in future multifunctional electronic applications.

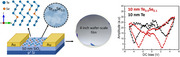

## 1 Introduction

Ferroelectric materials with noncentrosymmetric crystal structures can host spontaneous electric polarization and electromechanical coupling, enabling field‐tunable charge distribution and functional switching behaviors that are attractive for memory and neuromorphic electronics [1]. In CMOS‐relevant oxide platforms, polarization‐enabled device concepts are most commonly realized in engineered HfO_2_‐based dielectric stacks (e.g., doped or laminated HfO_2_ family films that stabilize a ferroelectric phase), which have received significant attention due to their compatibility with advanced semiconductor manufacturing [2, 3, 4, 5]. Nevertheless, oxide‐based ferroelectric dielectrics can face challenges related to ultrathin scaling, phase stability, and integration constraints, motivating continued exploration of alternative low‐dimensional ferroelectric materials that can operate at low voltage.

In this context, two‐dimensional (2D) van der Waals (vdW) ferroelectric materials offer a compelling route because polarization‐related functionalities can remain robust even when thinned toward the monolayer limit, while maintaining compatibility with standard Si integration processes [6, 7]. Their intrinsically non‐centrosymmetric lattices [8], weak interlayer interactions, and relatively clean surfaces can facilitate a stable electromechanical response and high‐quality interfaces in heterointegration [9]. In particular, out‐of‐plane polarization and piezoelectricity are favorable for high‐density integration and low‐power operation because they enable efficient electrostatic modulation and vertical‐field‐driven switching [10, 11]. As a result, polarization‐enabled phenomena in 2D materials have opened opportunities for multifunctional electronic applications [12], including nonvolatile memories, transistors, neuromorphic computing, and in‐memory processing [13]. Beyond vdW ferroelectric semiconductors, MXene‐based 2D systems have also been explored in polarization‐related contexts. For instance, T_3_C_2_T_x_ MXene films have been reported to exhibit hysteretic polarization‐like responses that can be sensitive to measurement conditions, such as frequency and electrical poling [14]. Moreover, MXenes can act as reactive precursors for deriving conventional ferroelectric compounds. However, translating MXene‐based polarization‐like behaviors into practical three‐terminal device operation typically requires careful control of materials chemistry and interfaces. Many MXenes are highly conductive, and their electronic properties and interfacial electrostatics are strongly influenced by surface terminations (T_x_) and adsorbates. Consequently, achieving reproducible field‐effect modulation and stable memory switching often demands additional control over surface chemistry, encapsulation, and processing conditions [15, 16].

Consequently, several 2D ferroelectric semiconductors, particularly those exhibiting n‐type transport, such as α‐In_2_Se_3_ [17, 18], MoS_2_ [19, 20], and CuInP_2_S_6_ (CIPS) [21, 22] have demonstrated stable out‐of‐plane polarization together with strong field‐effect modulation of carrier density, enabling polarization‐controlled transistors with memory switching behavior. Moreover, polarization in 2D materials provides additional functional advantages, including programmable doping [23] and dynamic modulation of interfacial properties through electrically induced lattice deformation [24]. However, compared with n‐type systems, the development of p‐type 2D ferroelectric semiconductors remains limited due to both intrinsic and extrinsic challenges [25]. A major limitation is the scarcity of intrinsic p‐type semiconductors that simultaneously exhibit stable out‐of‐plane polarization and sufficient hole mobility for device integration [26]. For example, although materials such as SnS and GeSe can show p‐type transport, their polarization switching under ultrathin scaling is often constrained by limited crystallinity, reduced polarization stability, and/or large switching fields [27, 28]. In addition, asymmetric charge trapping and interfacial screening in p‐type systems can destabilize the polarization states [29], complicating their implementation in memory and polarization‐enabled transistor architectures.

The recent theoretical prediction and experimental exploration of Tellurium (Te), which exhibits piezoelectricity with spontaneous polarization, with its helical chain structure and strong spin–orbit coupling [30], further highlight the versatility and fundamental significance in low‐dimensional systems [31]. Rao et al. fabricated a monolayer Te film using a hydrothermal method, which exhibits piezoelectric behavior and thermal stability [32]. This is pioneering research focused on the polarization behavior of elemental materials. Moreover, Zhang et al. explain the ferroelectricity of Te nanowires in detail through polarization direction and electrical measurements [33]. These advances position Te collectively as a key enabler in the development of multifunctional electronic and neuromorphic systems. Despite these advances, some issues remain in transforming the intrinsic properties of elemental Te fabricated by hydrothermal methods into practical device platforms. Although hydrothermal synthesis has enabled the formation of monolayer or nanowire forms of Te with high crystallinity and observable out‐of‐plane polarization, this method suffers from poor scalability, limited substrate compatibility, and low control over film uniformity. In addition, it is inherently unsuitable for large‐area or wafer‐scale integration required in modern electronic applications [34]. Therefore, identifying low‐temperature, scalable, and CMOS‐compatible growth strategies for Te‐based p‐type ferroelectric semiconductors is critical for unlocking their potential in next‐generation memory applications [35, 36]. The cryogenic physical vapor deposition (cryogenic PVD) has been demonstrated to stand out as a promising method due to its process controllability, substrate compatibility, and scalability [37, 38]. Moreover, the relatively low processing temperature (typically below 200°C) makes PVD highly compatible with back‐end‐of‐line (BEOL) CMOS processes, minimizing thermal stress on prefabricated device layers. However, piezoelectricity in cryogenic PVD‐grown Te films has not yet been systematically examined, due to the polycrystalline phase, which can complicate unambiguous polarization‐switching characterization. Therefore, creating a noncentrosymmetric crystal structure by adding other atoms in Te‐based films is an effective way to enhance the piezoelectricity of cryogenic PVD‐deposited Te‐based films.

In this regard, piezoelectric behavior in the 2D‐tellurium‐based family, namely 2D‐Te_x_Se_1‐x_, prepared by cryogenic PVD, is demonstrated and investigated in this work. By precisely adjusting the selenium (Se) content in the Te_x_Se_1‐x_ targets, the Se atomic ratio in the resulting Te_x_Se_1‐x_ thin films can be systematically controlled. Introducing Se atoms into 2D Te_x_Se_1‐x_ lattices promotes a higher degree of structural asymmetry [39], which strengthens the spontaneous polarization. Vertical piezoresponse force microscopy (PFM) was employed to probe the electromechanical switching behavior of the 2D‐Te_x_Se_1‐x_ thin films, where phase–voltage hysteresis and amplitude–voltage “butterfly” loops indicate a switchable electromechanical response that is consistent with polarization switching. Pronounced PFM‐based effective piezoresponse has also been reported in Te‐based thin films [40] and tellurene‐related systems [41], underscoring that such electromechanical signatures can be significant in this material family. Building on these prior observations, this work systematically investigates how growth temperature, crystallinity, and Se concentration modulate the PFM response in cryogenic PVD‐grown 2D‐Te_x_Se_1‐x_ alloys. Among the compositions studied, the Te_0.9_Se_0.1_ film exhibits the largest effective out‐of‐plane piezoelectric coefficient, with d_33_ reaching 33 pm/V under identical measurement conditions. Furthermore, the Te_0.9_Se_0.1_‐based memory device shows retention exceeding 2000 s and endurance up to 1000 cycles, with a well‐defined and stable memory window (HRS/LRS > 10^2^) under ± 20 V pulsed‐voltage triggering and read at a drain voltage of 1 V. Synaptic neural simulation has also been developed, achieving 92% accuracy in image recognition. The results provide an opportunity to integrate the Te_0.9_Se_0.1_ thin film with 3D IC circuits, paving the way for next‐generation memory designs.

## 2 Results and Discussion

### 2.1 Crystal Structures and Materials Characterizations of Te_x_Se_1‐x_ Thin Films

**FIGURE 1:**
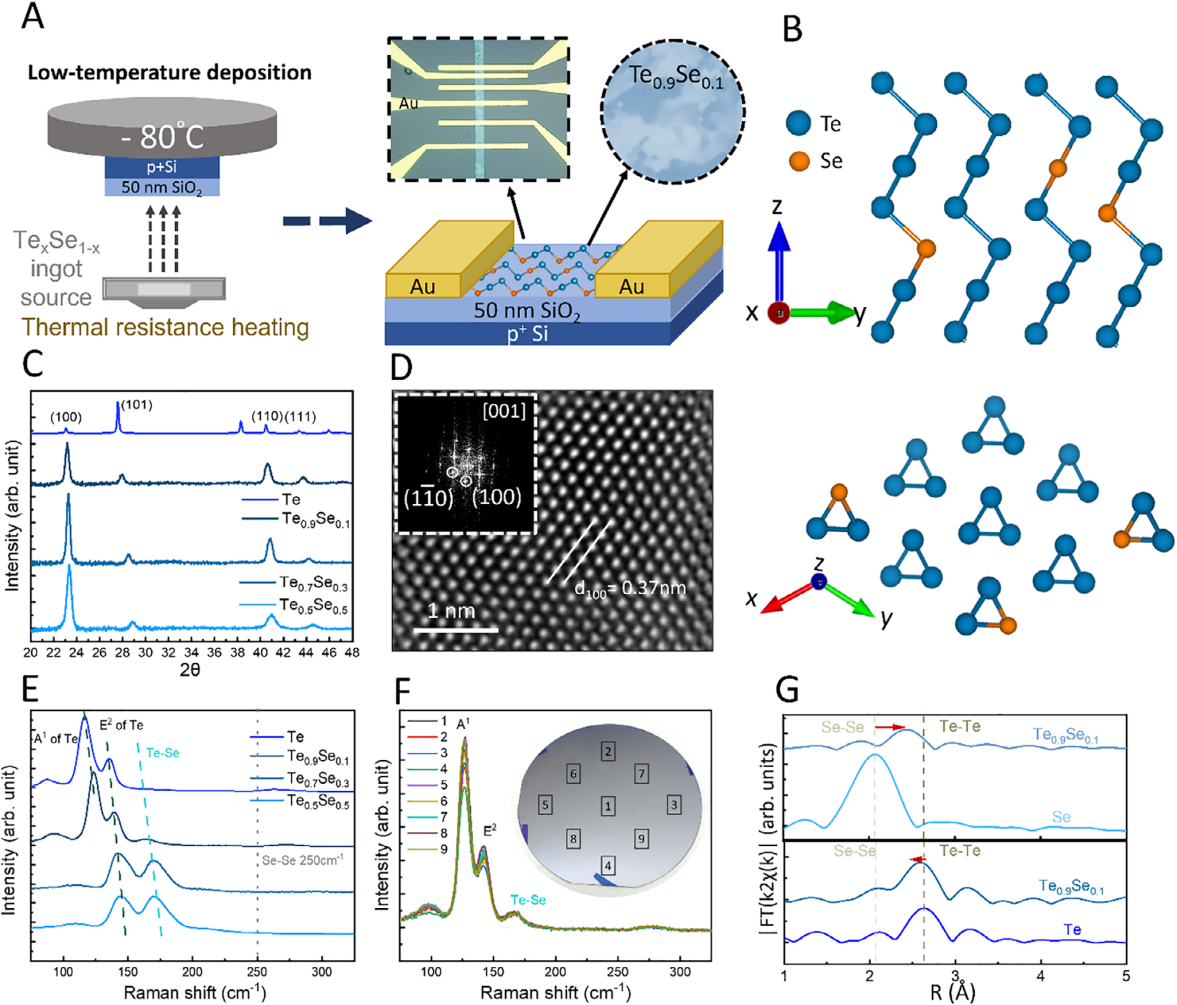
Crystal structure and materials characterization of Te_x_Se_1‐x_ thin films. (A) Schematic diagram of the synthesis of Te_x_Se_1‐x_ thin film‐based devices. Te_x_Se_1‐x_ shares the same helical chiral chain with Te. (B) (100) plane and (001) plane. (C) XRD patterns of Te_x_Se_1‐x_ thin films with different ratios. (D) STEM image of (001) plane Te_x_Se_1‐x_ thin film. (E) Raman spectrum of Te_x_Se_1‐x_ thin films with different ratios. (F) Raman spectra of a uniform Te_0.9_Se_0.1_ film deposited on a 4‐inch wafer. (G) Corresponding Fourier transform of Te K‐edge and Se K‐edge k^3^‐weighted EXAFS spectrum. The inset shows that the bonding length of Te_0.9_Se_0.1_ is between Te and Se.

Figure 1A shows schematic processes for the growth of 2D Te_x_Se_1‐x_ film by a cryogenic PVD method and device configuration. Figure S1A–D illustrates the preparation flow for 2D‐Te_x_Se_1‐x_ thin films for back‐gate devices, and the detailed growth conditions are described in Methods. Here, we found that the atomic ratios of the 2D Te_x_Se_1‐x_ thin films could be effectively controlled by metallurgically sintering Te_x_Se_1‐x_ precursor ingots from Te and Se powders with precisely tuned stoichiometry. The inset of Figure 1A displays a clearly visible polycrystalline 10 nm Te_0.9_Se_0.1_ thin film. Continuously, Figure 1B illustrates the trigonal phase of Te_x_Se_1‐x_, which shares the same chiral crystal structure as 1D Tellurium [42]. To precisely determine the space group of Te_x_Se_1‐x_, Electron Backscattered Diffraction (EBSD) was used to identify the preferred crystalline planes of the 2D‐Te_x_Se_1‐x_ thin film and its crystalline structure. The results show that Te_x_Se_1‐x_ shares the same space group as Te, and the target stoichiometry is consistent with the target source, as shown in Figure S2. To confirm the crystallinity, X‐ray diffraction (XRD) was performed on samples with different Te_x_Se_1‐x_ ratios, as shown in Figure 1C. Compared with the reference of pure Te, Te_x_Se_1‐x_ shows the same characteristic peaks, which correspond to plane (100), (101), (110), and (111), respectively. Calculated from Bragg's Law, the d‐spacing values for each plane are listed in Table S1. As the Se ratio increases, the d‐spacing values for each plane decrease due to the smaller radius of the Se atom compared to the Te atom [42]. Specifically, a plane view of the Scanning Transmission Electron Microscopy (STEM) image of the Te_0.9_Se_0.1_ thin film was obtained, as shown in Figure 1D, in which the diffraction pattern can be indexed along the (001) zone axis. Note that the d‐spacing of 0.37 nm was indexed along the (100) plane, which is also consistent with XRD results. Furthermore, Energy Dispersive Spectroscopy (EDS) confirms an atomic ratio of ∼89.9% Te and ∼10.1% Se (i.e., Te_0.9_Se_0.1_; Figure S3), while cross‐sectional TEM and EDS line scans verify a uniform Te/Se distribution throughout the film (Figure S4). In addition to structural characterization, the phase stability of Te–Se alloys needs to be considered. Prior equilibrium assessments and the Se–Te phase diagram indicate that Se_1‐x_Te_x_ is commonly described as a solid‐solution system with favorable mixing behavior and no pronounced thermodynamic driving force for spinodal decomposition over the relevant composition range under equilibrium conditions [43]. In the cryogenic PVD process (at −80°C), the low growth temperature further limits atomic mobility and kinetically suppresses any long‐range diffusion required for composition demixing. Consistent with this expectation, EBSD verifies a trigonal Te‐type phase with the target stoichiometry (Figure S2), while cross‐sectional EDS line scans confirm a uniform Te/Se distribution without detectable segregation (Figure S4).

Figure 1E demonstrates Raman spectra of 2D‐Te_x_Se_1‐x_ thin films with different Se atomic ratios. The Raman spectra of the pure Te exhibit two peaks at 123.1 and 142.2 cm^−1^, which are related to A^1^ and E^2^ vibration modes, respectively [37]. As the atomic ratio of Se increases, the A^1^ mode disappears, while the E^2^ mode gradually shifts to higher wavenumbers, since the Se–Se vibrational mode is located at 250 cm^−1^ [39]. More specifically, the emergence of a new vibrational mode around 175 cm^−1^, corresponding to Te─Se bonding, provides evidence that the degree of noncentrosymmetry increases as Se atoms substitute for Te at specific lattice sites. Furthermore, the cryogenic PVD method yields Te_0.9_Se_0.1_ thin films with good large‐area uniformity, as evidenced by the consistent and continuous Raman spectra collected from different locations across the film (Figure 1F). To further investigate the bonding environment influenced by Se incorporation, extended X‐ray absorption fine structure (EXAFS) analysis was conducted. The corresponding Fourier transform of the Te K‐edge and Se K‐edge k^3^‐weighted EXAFS spectra of the Te_0.9_Se_0.1_ thin film are shown in Figure 1G. The characteristic bonding lengths of Se and Te reference are calculated to be 2.1 Å and 2.6 Å [44], which are longer than that of pure Se but shorter than that of pure Te. This confirms that the incorporation of Se introduces local structural disorder and bonding randomness into the Te_0.9_Se_0.1_ lattice. The composition‐dependent EXAFS results in Figure S5A,B provide additional evidence. This local structural disorder and randomness observed in Te_x_Se_1‐x_ thin films relative to pure Te can be a key factor to explain the non‐centrosymmetry introduced by Se atoms, suggesting enhanced suitability for piezoelectric‐related spontaneous polarization functionality.

### 2.2 PFM Analysis of Piezoelectric Property

**FIGURE 2:**
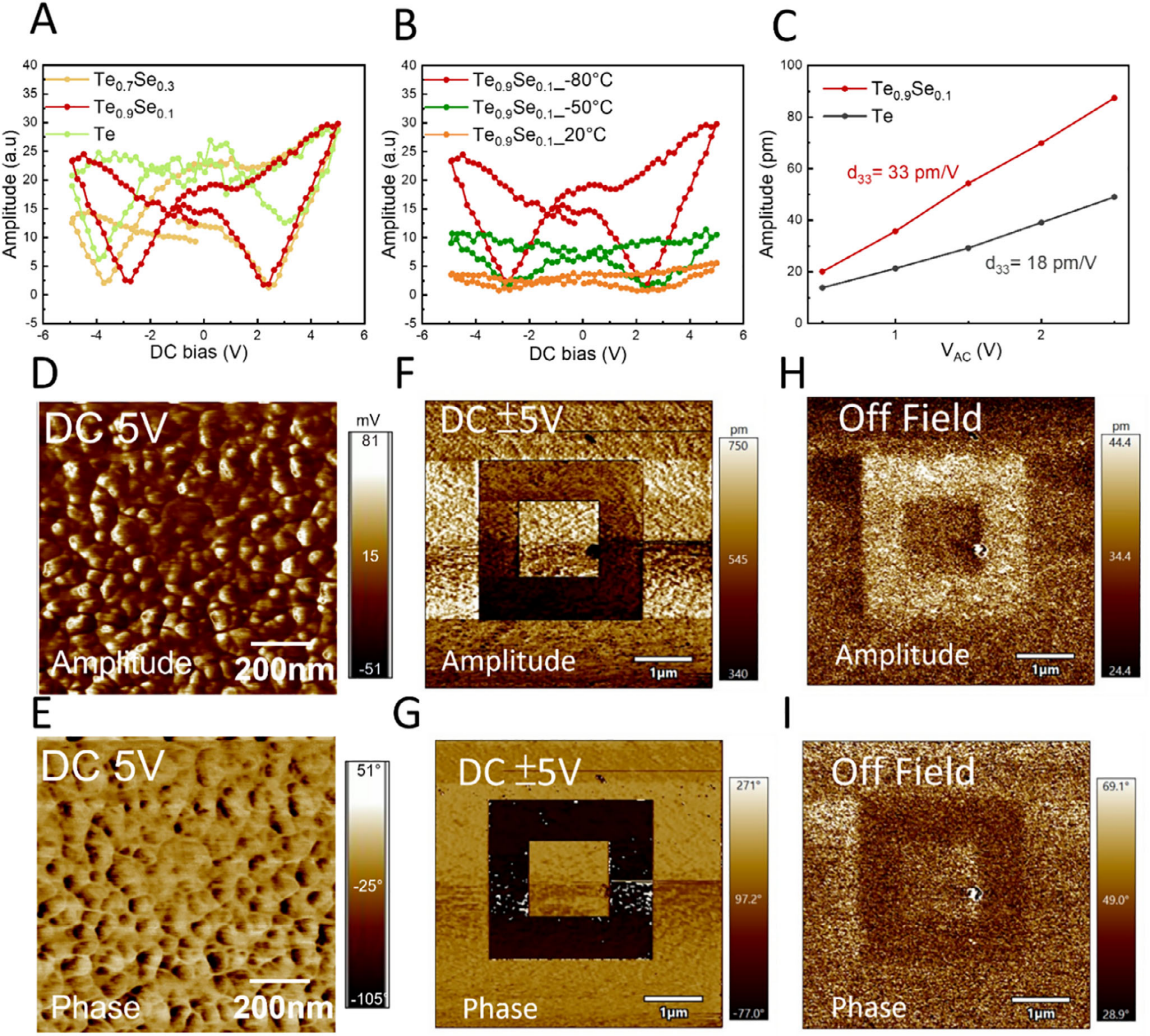
PFM analysis of piezoelectric behaviors (A) Off‐field amplitude–voltage butterfly loops of 10 nm Te_x_Se_1‐x_ with different ratios. (B) Off‐field amplitude–voltage hysteresis loops of 10 nm Te_0.9_Se_0.1_ with different deposition temperatures. (C) The piezoelectric coefficient (d_33_) of pure Tellurium and Te_0.9_Se_0.1_. (D) Response of PFM amplitude, which shows the piezoelectric domain in Te_0.9_Se_0.1_ thin film with V_DC_ = 5 V. (E) Response of the PFM phase, which shows a piezoelectric domain in the Te_0.9_Se_0.1_ thin film with V_DC_ = 5 V. (F) Response of PFM amplitude of Te_0.9_Se_0.1_ thin film with V_DC_ = ± 5 V (On‐Field). (G) Response of the PFM phase of the Te_0.9_Se_0.1_ thin film with V_DC_ = ± 5 V (On‐Field). (H) Response of PFM amplitude of Te_0.9_Se_0.1_ thin film after removing V_DC_ (Off‐Field). (I) Response of the PFM phase of Te_0.9_Se_0.1_ thin film after removing V_DC_ (Off‐Field).

The Piezoelectric Force Microscopy (PFM) was conducted to address the piezoelectric effect enhanced by the incorporation of Se atoms into Te_x_Se_1‐x_ thin films. The PFM is a nondestructive imaging and manipulation method for observing piezoelectric electromechanical domains at the nanoscale [45]. Spontaneous polarization associated with the piezoelectricity offers a promising route for memory device fabrication. To further examine the piezoelectric response, off‐field phase‐voltage hysteresis loops and amplitude‐voltage butterfly loops with different ratios were measured by vertical PFM (Figure 2A and Figure S6A). Off‐field piezoresponse hysteresis loops were employed to investigate the intrinsic piezoelectric properties by minimizing electrostatic artifacts [19]. Compared to the pure Te thin film, the Te_0.9_Se_0.1_ thin film exhibits well‐defined and stable phase and amplitude hysteresis loops, indicating more pronounced and characteristic polarization switching behavior. However, when the Se content increases to 0.3, the loops become distorted, attributed to a decrease in crystallinity. Note that the deposition temperature also plays a critical role in determining the crystallinity of the Te_0.9_Se_0.1_ thin film. The comparison results of off‐field amplitude‐voltage butterfly loops of the 10 nm Te_0.9_Se_0.1_ thin film deposited at −80°C, −50°C, and 20°C are shown in Figure 2B, respectively. The DC bias corresponding to the local minimum of the amplitude response reflects the effective coercive voltage (V_c,eff_) of the material [46], which can be observed to be approximately ± 2 to ± 3 V for the Te_0.9_Se_0.1_ film. The corresponding phase–voltage hysteresis loops are demonstrated in Figure S6B. The amplitude responses of piezoelectric behavior in the Te_0.9_Se_0.1_ thin film become more pronounced at low deposition temperature, where the phase loop exhibits clearer, more defined hysteresis. The results verify that grain size plays an important role in reversing polarization. To support this evidence, optical microscopy (OM) images of different grain sizes in Te_0.9_Se_0.1_ thin films can be found at different deposited temperatures from −80 to 20°C (Figure S7A–C). The largest grain size of the Te_0.9_Se_0.1_ is achieved at the deposited temperature of −80°C. Figure S7D–F present OM images of the 10 nm Te_x_Se_1‐x_ thin films with different ratios at the deposited temperature of −80°C, revealing different grain sizes. The smaller grains can be attributed to enhanced heterogeneous nucleation induced by Se incorporation [39], which may also contribute to the reduced piezoelectric response at higher Se content (Figure 2A). Overall, the optimal largest grain size can be found in the Te_0.9_Se_0.1_ film at the deposited temperature of −80°C.

The effective out‐of‐plane piezoelectric coefficient d_33_ is extracted from the linear regime of the average PFM amplitude as a function of the AC driving voltage (V_AC_). To minimize field‐driven switching and electrostatic/charge‐injection contributions during quantification, V_AC_ was chosen to remain well below the loop‐opening,V_c,eff_, defined here as the characteristic voltage scale for hysteresis observed under the specific tip‐sample geometry. When V_AC_ approaches V_c,eff_, the measured amplitude can deviate from linearity and exhibit a slight reduction, which is consistent with the onset of non‐linear switching dynamics and enhanced electrostatic interactions under strong local fields. It is crucial to restrict the d_33_ determination to the low‐V_AC_ linear region and use this procedure to compare compositions under identical measurement conditions. While this trend is consistent with a switchable electromechanical response, tip‐based PFM signals can be influenced by nonpiezoelectric effects, such as electrostatic contributions and charge injection. Therefore, the piezoelectric behavior is evaluated in conjunction with complementary controls and device‐level nonvolatile characteristics [33, 47]. Therefore, the average amplitudes under varying V_AC_ for Te and Te_0.9_Se_0.1_ thin films are presented in Figure S8, varying V_AC_ from 0.5 to 2.5 V. The summarized results are depicted in Figure 2C, from which the extracted d_33_ values are 18 pm/V and 33 pm/V for Te and Te_0.9_Se_0.1_ thin films, respectively. It verifies the existence of an electric dipole moment and stronger piezoelectricity in the Te_0.9_Se_0.1_ thin film. Furthermore, the PFM amplitude and phase responses obtained over a 1 µm × 1 µm scanning area reveal uniform piezoelectric domains in the Te_0.9_Se_0.1_ thin film under an applied bias of V_DC_ = 5 V, as shown in Figure 2D,E. The results confirmed that the piezoelectric behavior of the spontaneous polarization is clear in each grain. The PFM amplitude and phase responses of Te_0.9_Se_0.1_ thin film obtained over a 5 µm × 5 µm scanning area with V_DC_ = ± 5 V lithography pattern (On‐Field) are shown in Figure 2F,G. The apparent difference in amplitude and the phase contrast exceeding 180° illustrate the piezoelectric effect under opposite applied voltages. To evaluate the retained domain contrast after poling, the PFM amplitude and phase were recorded after removing V_DC_ = ± 5 V (off‐field), as shown in Figure 2H,I. The remaining contrast of the area shows good retention capability. Moreover, long‐time off‐field PFM amplitude and phase images are collected in Figure S9 to test the stability of the piezoelectric effect under ambient conditions. The sustained retention over 20 min indicates that the observed PFM contrast originates from intrinsic piezoelectric behavior rather than transient or extrinsic artifacts.

Consequently, to further examine the spontaneous polarization behavior, symmetric metal/semiconductor/metal (MSM) capacitors (Au/Te_0.9_Se_0.1_/Au) were fabricated (Figure S10A). Positive‐Up/Negative‐Down (PUND) measurements were performed, and the schematic of the triangular input pulse sequence (pulse width: 5 ms) and the corresponding pulse‐resolved I–t transient curve are shown in Figure S10B. The amplitude‐dependent polarization–electric field (P‐E) characteristics (Figure S10C) were measured over an electric field range of ± 0.5 V/nm with pulsed widths of 5 ms and 10 ms, and the current–electric field (I–E) hysteresis loop is shown in Figure S10D. The polarization curves measured at two different pulse widths show similar behavior, indicating that the extracted polarization response is reproducible across pulse‐width conditions. The measured remanent polarization values are P_r_^+ ^= 0.086 µC/cm^2^ and P_r_^− ^= ‐0.068 µC/cm^2^, respectively. Although the P–E response is not fully saturated within the applied field range, the reproducible hysteresis and finite remanent polarization extracted from PUND under two pulse widths (5 and 10 ms) suggest a polarization‐related switching trend in the Te_0.9_Se_0.1_ film. In addition, capacitance–voltage (C–V) measurements of the same Au/Te_0.9_Se_0.1_/Au capacitor measured at 1 MHz are shown in Figure S10E. The capacitance of Te_0.9_Se_0.1_ film is around ∼0.6 pF. In summary, polarization switchability in Te_0.9_Se_0.1_ is evidenced by the combined PFM piezoresponse hysteresis and PUND measurements. Also, this work is not to overstate the presence of a fully established macroscopic ferroelectric characteristic, but rather to present the current results as supportive evidence of polarization‐related switchability in Te_0.9_Se_0.1_.

### 2.3 Hysteresis Loop and Assessments of Memory Behaviors

Considering that pure Te exhibits a p‐type semiconducting behavior [37, 38], its inherent vertical spontaneous polarization enables pure Te to serve as a potential channel material in FETs. However, multilayer Te has a narrow bandgap of ∼0.3 eV, leading to a low on/off current ratio and elevated leakage current. By modulating the Se content in Te_x_Se_1‐x_ thin films, the bandgap can be effectively tuned [39, 44, 48], thereby improving transistor performance. Figure S11 illustrates the transfer characteristics I_d_‐V_g_ of Te_x_Se_1‐x_‐based FETs under various deposited temperatures and thicknesses. Te_0.9_Se_0.1_ thin films crystallized at −80°C and −50°C exhibit significantly enhanced current modulation compared to the noncrystallized counterpart, indicating that the improved crystallinity promotes more efficient carrier transport and reduces leakage current (Figure S11A). In addition, among Te_0.9_Se_0.1_ thin films with different thicknesses (Figure S11B), the 10 nm Te_0.9_Se_0.1_ demonstrates optimal transfer characteristics. However, the thinnest Te_0.9_Se_0.1_ (5 nm) shows degraded current modulation, likely due to insufficient continuity or increased surface scattering. These results collectively confirm that the Te_0.9_Se_0.1_ thin film, with proper crystallization (−80°C) and optimized thickness (∼10 nm), is crucial for achieving high‐performance Te_x_Se_1‐x_‐based applications.

**FIGURE 3:**
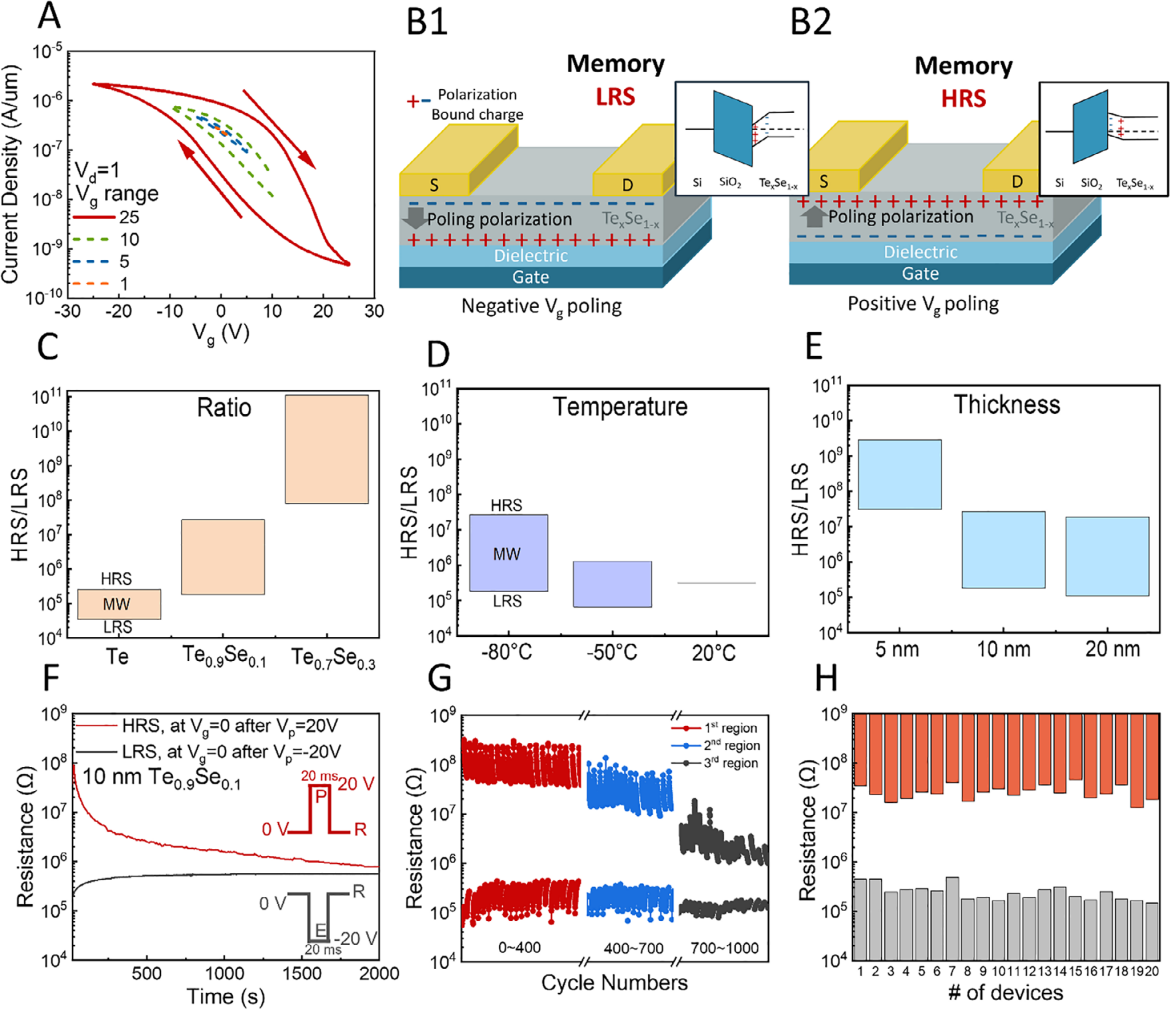
Measurements of hysteresis loop and memory behaviors. (A) Hysteresis transfer characteristics of Te_0.9_Se_0.1_ thin film‐based FET with gate voltage ranging from 25 V to −25 V, 10V∼‐10 V, 5V∼‐5 V, and 1V∼‐1 V. The drain voltage is set as 1 V, and the direction of the hysteresis loops shows clockwise. Schematic diagram illustrating the working principle of the p‐type Te_0.9_Se_0.1_‐based memory functionality under an external electric field. (B1) After negative gate‐bias poling, positive bound charges are induced at the bottom side of the channel and negative bound charges at the top side, which favors a lower‐resistance state (LRS). (B2) After positive gate‐bias poling, the bound‐charge distribution is reversed, leading to reduced hole density near the bottom interface and a higher‐resistance state (HRS) (C) The Memory window of 10 nm Te_x_Se_1‐x_ with different ratios measured by the memory test. (D) The Memory window of 10 nm Te_0.9_Se_0.1_ with different deposition temperatures measured by the memory test. (E) The Memory window of Te_0.9_Se_0.1_ with different thicknesses measured by the memory test. (F) Quasi‐nonvolatile memory retention characteristics of Te_0.9_Se_0.1_, with program/erase pulsed voltage of ± 20 V and a pulse width of 10 ms, followed by read‐after‐delay at V_g_ = 0 V and V_d_ = 1 V, retention time over 2 × 10^3^ s. (G) Endurance characteristics of the Te_0.9_Se_0.1_ thin‐film–based memory device measured up to 1000 program/erase (write/erase) cycles. The HRS/LRS ratio exceeds 10^2^ in the first region (0–400 cycles), decreases to ∼ 10^2^ in the second region (400–700 cycles), and shows pronounced degradation in the third region (700–1000 cycles), where the HRS/LRS ratio drops to ∼10. (H) Memory characteristics tests with 20 devices.

Next, the hysteresis transfer characteristics of 10 nm Te_0.9_Se_0.1_ thin film‐based FETs were evaluated under gate voltage sweeps ranging from ± 25, ± 10, ± 5, and ± 1 V, with a constant drain voltage of 1 V, as shown in Figure 3A. The clockwise hysteresis observed in our p‐type devices is also consistent with the expected electrostatic modulation associated with spontaneous polarization. Since clockwise I_d_–V_g_ hysteresis can also originate from other processes, including charge trapping [49], ionic gating [50], and environmental dipolar/adsorbate effects recently reported in ultrathin Te transistors by Wang et al. [51]., additional measurement conditions and control experiments are provided in Note S1 and Figure S12 to examine these alternative contributions. In particular, Wang et al. showed that the dynamic reorientation of surface‐adsorbed gas molecules can govern pronounced hysteresis in exposed Te transistors. In contrast, dielectric encapsulation suppresses this gas‐induced component, revealing a remaining charge‐trapping contribution in single‐gate devices. While such extrinsic effects may contribute to the overall hysteresis response, the combined results support a polarization‐related contribution beyond a purely trap‐ or environment‐dominated interpretation in the present Te_x_Se_1−x_ devices. Also, the long‐term stability of the device after one year is presented in Figure S12D; the on/off ratio and hysteresis characteristics remain unchanged, indicating stable device performance over time. To ensure consistency, over 20 devices were measured and showed clockwise hysteresis in the I_d_–V_g_ loop, indicating film uniformity and potential for large‐scale application (Figure S13A). In addition to the device utilizing a 50 nm SiO_2_ dielectric layer, 10 nm Te_0.9_Se_0.1_ thin films were also fabricated on a 10 nm ZrO_2_/Al_2_O_3_/ZrO_2_ (ZAZ) dielectric layer to demonstrate the reproducibility of polarization switching behavior on different substrates, as shown in Figure S13B. The clockwise hysteresis direction was also observed in the device with the ZAZ dielectric layer, exhibiting a steeper subthreshold slope and reduced leakage current. The detailed operating mechanism of the p‐type memory device is illustrated in Figure 3B1,B2. Following gate poling, polarization‐induced bound charges are established at the opposite sides of the Te_x_Se_1−x_ channel, generating an internal electrostatic field that modulates the hole distribution across the channel thickness. In the back‐gated device geometry, however, the conductance state is determined predominantly by the electrostatic potential near the bottom Te_x_Se_1−x_/SiO_2_ interface, because this is the region most directly coupled to the gate field and most relevant to the effective source‐to‐drain transport path. Therefore, the measured resistance switching mainly reflects accumulation or depletion of holes in the interfacial channel region adjacent to the dielectric. After negative gate‐bias poling, positive bound charges are induced at the bottom of the channel and negative bound charges at the top, which favors a lower‐resistance state (LRS) (Figure 3B1). After positive gate‐bias poling, the bound‐charge distribution is reversed, leading to reduced hole density near the bottom interface and a higher‐resistance state (HRS) (Figure 3B2). This mechanism is consistent with polarization‐mediated electrostatic control of interfacial channel transport in back‐gated 2D memory devices [52].

To be more specific, a gate voltage of ± 20 V (± 2 V for the ZAZ substrate) with a 10 ms pulsed width was used to confirm the memory retention characteristics of the Te_x_Se_1‐x_ thin film. Quasi‐nonvolatile memory retention characteristics of the Te_x_Se_1‐x_ films with different ratios, different deposition temperatures, and different thicknesses are shown in Figures S14–S16, respectively. In addition, the quasi‐nonvolatile memory retention of 10 nm Te_0.9_Se_0.1_ thin film with the ZAZ layer as the gate dielectric layer is shown in Figure S13C. The summarized results for HRS/LRS are presented in Figure 3C–E. As the Se concentration increases, the overall resistance of the device rises, which can be attributed to changes in crystallinity (Figure 3C and Figure S14). Simultaneously, the enlarged memory window (HRS/LRS) can be achieved due to the enlarged band gap associated with the higher Se concentration. Regarding the deposition temperature, Te_0.9_Se_0.1_ thin films deposited at −80°C exhibit the widest memory window because of larger polarization domains, which can be attributed to the reduced grain boundary density (Figure 3D and Figure S15). However, varying the thickness of Te_0.9_Se_0.1_ thin films has the opposite effect on the memory window, with the primary difference being an increase in resistance observed in the 5 nm Te_0.9_Se_0.1_ thin film. In brief, based on the transistor performance results presented in Figure 3E and Figure S16, the 10 nm Te_0.9_Se_0.1_ thin film demonstrates optimal characteristics, including enhanced current modulation, a wider memory window, lower leakage current, and clearer polarization switching behavior, making it the most suitable parameter for application in both transistor and memory devices. The resistance–pulsed‐voltage hysteresis loops of 10 nm Te_0.9_Se_0.1_ film measured at pulse durations of 0.1 ms, 1 ms, and 20 ms are shown in Figure S17. Similar resistive states are obtained across these pulse‐width conditions, indicating that the switching behavior is reproducible and not strongly dependent on the pulse duration within the tested range. Notably, even a short pulse width of 0.1 ms is sufficient to switch the resistance state, supporting the pulse‐induced switching behaviors. Besides, the 10 nm Te_0.9_Se_0.1_ thin film exhibits distinguishable retention under ambient conditions, exceeding 2000 s, as shown in Figure 3F. The HRS and LRS gradually relax toward the initial state, resulting in a reduced memory window when the time is prolonged to over 1000s. This behavior is consistent with environmental/interfacial effects commonly observed in 2D‐channel devices, where adsorbates (notably moisture) and surface screening/interfacial charge equilibration can partially compensate for the polarization‐induced field effect and accelerate state relaxation [53]. Further encapsulation and controlled‐environment measurements will be required to evaluate long‐term retention stability. In addition, the endurance characteristics up to 1000 program/erase cycles are presented in Figure 3G. The HRS/LRS ratio remains above 10^2^ through the first 400 cycles and remains around 10^2^ through approximately 700 cycles. At higher cycling numbers, clear degradation of the conductance window is observed, and the HRS/LRS ratio decreases to approximately 10 by 1000 cycles.

To elucidate the origin of this degradation, post‐cycling analyses were performed using OM, cross‐sectional TEM, and EDS line scans (Figure S18). After 1000 cycles, OM images reveal visible damage near the channel–electrode region (Figure S18A,B). Consistently, the cross‐sectional TEM shows a clear and smooth channel/electrode interface before cycling (Figure S18C), whereas the interface becomes blurred after cycling (Figure S18D). The EDS line‐scan profiles further suggest Au diffusion from the electrodes into the Te_0.9_Se_0.1_ channel after cycling (Figure S18E,F), which may reduce the channel resistance and could primarily lower the HRS, thereby shrinking the memory window. This observation is consistent with prior reports of metal diffusion into Te‐based channels in Te FETs after thermal/optical annealing [54], which have been discussed as potentially related to the relatively low thermal stability of Te‐based materials [55]. Accordingly, Au diffusion is considered a plausible contributor to the observed endurance degradation in our devices. In addition, the reproducibility of the switching behavior is supported by multi‐device endurance measurements, showing stable operation over 700 cycles (Figure S19A–D), with the corresponding HRS/LRS statistics and error bars summarized in Figure S19E. To assess the device uniformity and scalability, measurements were conducted on 20 individual memory devices, revealing consistent performance across samples, as illustrated in Figure 3H. As illustrated in Figure 3B1,B2, applying a negative gate voltage (V_g_) induces hole accumulation at the channel top, thereby reducing channel resistance (Figure S20A). Conversely, a positive V_g_ depletes holes in the channel, increasing channel resistance (Figure S20B). This behavior enables the realization of multiple resistance states, depending on the magnitude of the applied different V_g_. These results collectively highlight the potential of Te_x_Se_1‐x_ thin films for next‐generation nonvolatile memory devices.

### 2.4 Evaluation of Synaptic Neural Behaviors and Energy Consumption

**FIGURE 4:**
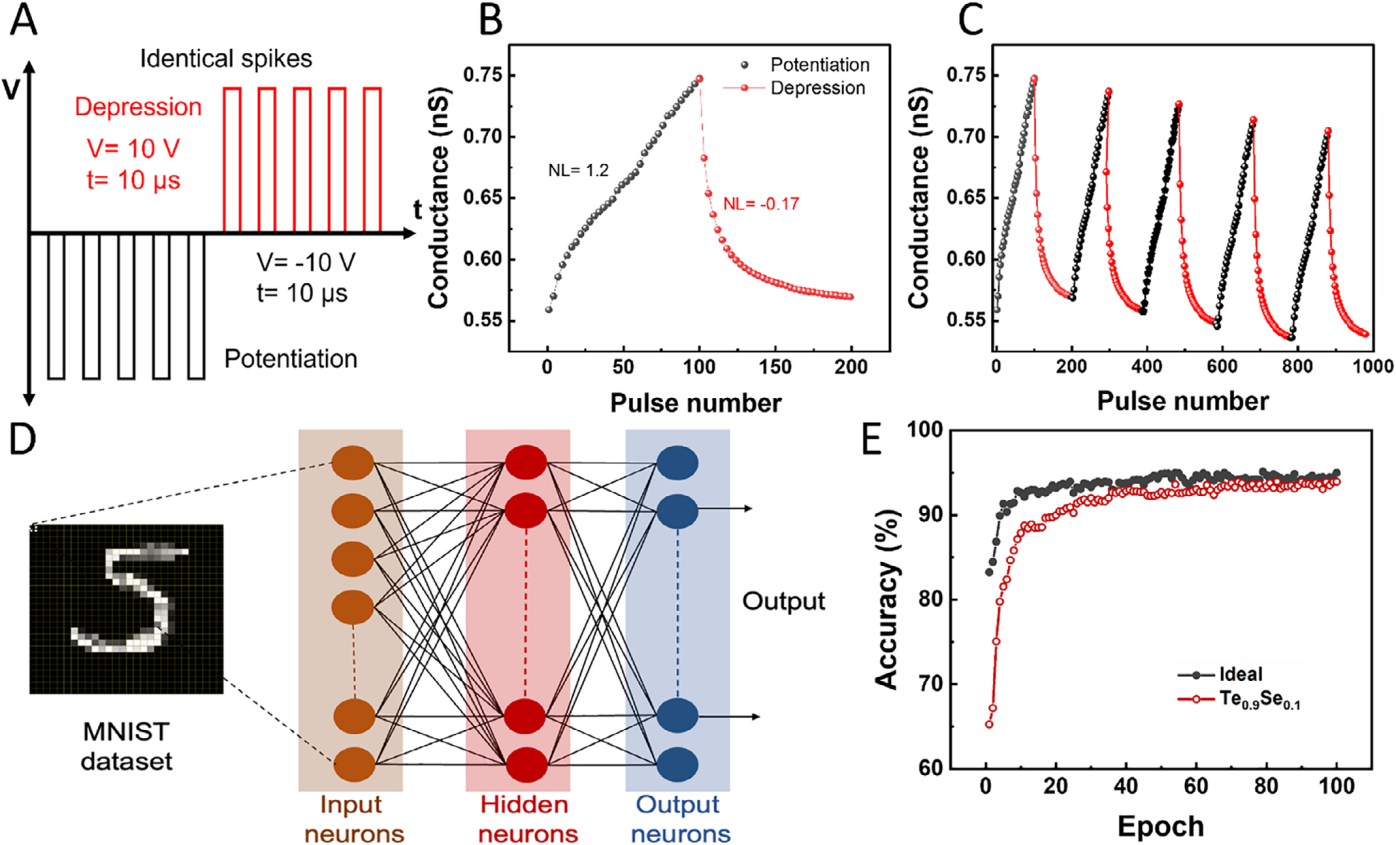
Simulation of the synaptic neural behavior by the Te_0.9_Se_0.1_ thin film‐based FET device. (A) Tunable potentiation and depression by applying an identical pulse with a pulse width of 10 µs and a pulse amplitude of ± 10 V. (B) Nonlinearity values for long‐term potentiation (LTP) and long‐term depression (LTD). (C) Conductance values of LTP and LTD were obtained by applying 30 consecutive positive and negative pulses. (D) A three‐layer convolutional neural network (CNN) to emulate online/offline learning using a handwritten dataset. (E) Image recognition accuracy in training a Te_0.9_Se_0.1_ thin film‐based FET device.

Inspired by the 10 nm Te_0.9_Se_0.1_ thin film‐based FET and memory characteristics discussed above, the synaptic neural behavior has been investigated. Short‐term plasticity (STPL) and long‐term plasticity (LTPL) serve as fundamental mechanisms for brain‐inspired computing and pattern recognition. Within the LTPL, long‐term potentiation (LTP) and long‐term depression (LTD) are critical prerequisites for enabling memory functionality in artificial synaptic devices. Similarly, LTP/LTD profiles can be mimicked in Te_0.9_Se_0.1_‐based devices. Figure 4A,B illustrates the characteristics of LTP and LTD in the fabricated FET devices. The periodic square wave pulses with uniform width and amplitude were applied. For potentiation, the device is initially subjected to identical negative gate voltage pulses (−10 V/10 µs) set to the depleted state of the Te_0.9_Se_0.1_ channel, followed by a series of identical negative gate voltage pulses (−10 V/10 µs). Conversely, for the depression cycle, the device starts from the accumulated state and is subjected to a sequence of uniform positive gate‐voltage pulses (10 V, 10 µs). After each pulse, the conductance was measured in the absence of the gate bias to evaluate the non‐volatile conductance modulation. The device shows linear conductance variation with a nonlinearity value of (1.2/−0.17), which is accurate for pattern recognition. Achieving high linearity and symmetric LTP/LTD characteristics is essential for reliable performance in neuromorphic computing and pattern recognition tasks [56]. The description of the weight update calculation is provided in Note S2. The conductance of the devices gradually increases (decreases) for consecutive 100 positive (negative) pulses, corresponding to symmetric LTP and LTD behaviors. Furthermore, the device shows stable LTP/LTD changes for five consecutive cycles, as shown in Figure 4C. Based on the measured LTP and LTD characteristics in our device, we have simulated an artificial neural network (ANN) to perform image recognition with the Modified National Institute of Standards and Technology (MNIST) handwritten data set [57]. The neuromorphic demonstration employs off‐chip simulation by mapping experimental LTP/LTD characteristics to synaptic weights in a software neural network [58]. For the simulation, an ANN network with 400 input, 100 hidden, and 10 output neurons, using a passive cross‐bar array architecture, was constructed, as shown in Figure 4D. The artificial neural network (ANN) was configured with 400 input neurons corresponding to 20 × 20‐pixel MNIST images, and 10 output neurons representing the digit classes from 0 to 9. During training, the simulator randomly selects samples from a training dataset of 60 000 images and evaluates recognition accuracy using a separate test set of 10 000 images. After 100 training epochs, the network achieves a maximum recognition accuracy of approximately 92%, comparable to the ideal software baseline (93%), as shown in Figure 4E. The experimentally observed endurance degradation becomes noticeable only after extended cycling, typically approaching **∼**700–1000 switching cycles and near device failure around 10^3^ cycles; therefore, such high‐cycle degradation is not expected to materially affect the simulation results obtained under the 100‐epoch training condition. Accordingly, a cycle‐dependent degradation model, including conductance‐window collapse at high cycling numbers, was not incorporated into the present simulation, which is intended strictly as a proof‐of‐concept evaluation of learning feasibility based on the measured synaptic characteristics. The obtained off‐chip recognition accuracy of 92% is therefore presented only as a simulation‐level result, rather than as a reliability‐aware hardware benchmark. Although this value is comparable to those reported for many 2D material systems (92%–97%) [59, 60, 61] and oxide memristors (95%–98%) [62, 63, 64], it also indicates that further improvements in linearity, variability, and cycling stability will be required for practical hardware implementation. Taken together, these results provide an initial indication that the measured Te_0.9_Se_0.1_ synaptic characteristics can support proof‐of‐concept neuromorphic simulations, while further work is still needed to assess long‐term reliability and hardware‐level performance.

2D ferroelectric materials, with their atomically thin nature, offer the potential for ultra‐high integration densities and low power consumption [6]. To assess the low power consumption of 2D Te_0.9_Se_0.1_‐based FETs, the energy consumption per spike of simulated synapses/neurons was calculated by $E=\frac{1}{2}\;C_{g}{V_{g}}^{2}+\int_{0}^{T}I_{d}\times V\;dt≅\;\frac{1}{2}C_{g}{V_{g}}^{2}+I_{d}\times t_{w}\times V$, which represents gate programming energy of transistor and synapses/neurons programming energy separately [65]. In this expression, C_g_ and V_g_ denote the gate‐oxide capacitance and the applied gate voltage, respectively. I_d_ and t_w_ represent the drain‐current response and the spike (pulse) duration, and V refers to the source–drain voltage in three‐terminal devices [66]. Here, the energy consumption of 2D Te_0.9_Se_0.1‐_based FETs for neuromorphic computing, along with comparisons of key characteristics of n‐type and p‐type 2D ferroelectric devices, is presented in Table 1. To date, research on 2D ferroelectric semiconductors remains relatively limited, especially when compared to the extensively studied insulators. Among the various n‐type 2D semiconductors investigated, MoS_2_ stands out for its high carrier mobility, as demonstrated experimentally in high‐current devices. InSe, on the other hand, exhibits stable polarized properties, making it a promising channel material for future low‐power electronic applications [67]. However, developing p‐type semiconductors with intrinsic piezoelectric properties remains a significant challenge. In this work, a 2D p‐type Te_0.9_Se_0.1_ thin film can be synthesized via low‐temperature deposition, enabling large‐scale integration. Besides, using a 50 nm SiO_2_ gate stack, the average memory window (HRS/LRS) is about 10^2^  via a write voltage of ± 20 V (read at V_g_ = 0, and V_d_ = 1 V). Also, a 10 nm ZAZ dielectric can act as a high‐𝜅 gate oxide, enabling a memory window of ∼50 with a low write voltage of ± 2 V (read at V_g_ = 0, and V_d_ = 1 V). This demonstrates clear voltage scaling enabled by the high‐𝜅 gate stack compared with conventional SiO_2_‐gated devices, while maintaining a measurable quasi‐nonvolatile resistance contrast. With the continuous scaling of gate oxides to advanced technology nodes, high‐κ dielectric gate oxides may face concerns about die scale reduction and memory window shrinkage [68]. In pursuit of low‐power applications within advanced CMOS technology, semiconductor materials with low switching voltages are highly desirable, as the write voltage must remain below the logic‐compatible threshold of 1.2 V to ensure on‐chip integration compatibility [19, 69]. Although the present ± 2 V operation is still above this 1.2 V target, it represents an important step toward logic‐compatible operation, and further scaling of the equivalent oxide thickness and device geometry is expected to reduce the required write voltage. In neuromorphic devices, Te_0.9_Se_0.1_ thin film demonstrates relatively low energy consumption, positioning it as a leading candidate among p‐type materials. When integrated with ultra‐scaled gate dielectrics, Te_0.9_Se_0.1_ thin film still maintains a distinct memory window under low supply voltages, making it a promising candidate for low‐power nonvolatile memory and logic device applications.

**TABLE 1:** Comparison of key characteristics of n‐type and p‐type 2D ferroelectric devices.

	Type	t_FE_ (nm)	Energy consumption (J)	Memory window (HRS/LRS)	Writing voltage (Field V/nm)	Read conditions (V)	Pulse width (s)	Endurance	Retention (s)	Year	References
InSe	n‐type 2D	14	N/A	10^5^	± 80 (0.27)	V_g_ = 0, V_d_ = 0.1	10^−1^	>10^2^	>10^3^	2020	[17]
ST‐3R MoS_2_	n‐type 2D	1.3	N/A	10^2^	± 10 (0.25)	V_g_ = 0, V_d_ = 1	10^−6^	>10^4^	>10^4^	2023	[19]
MoTe_2_	n‐type & p‐type 2D	2‐11	7 × 10^−16^ ^a^	10^3^	−3 (0.03)	V_g_ = ‐3, V_d_ = 0.1	10^−1^	>10^2^	10^5^	2023	[70]
Te nanowire	p‐type 2D	22	N/A	10^2^	± 100 (1.1)	V_g_ = 0, V_d_ = 0.5	10^−5^	>10	10^5^	2024	[33]
Te_0.9_Se_0.1_ thin film	p‐type 2D	10	6.8 × 10^−12^ ^b^	2×10^2^, 5×10^1^	± 20, ± 2 (0.4, 0.2)	V_g_ = 0, V_d_ = 1	10^−5^	>10^3^	>2 × 10^3^	This work	

^a^ The energy consumption is calculated by multiplying the lowest modulatory voltage by the corresponding modulatory current.

^b^ The energy consumption is calculated by $\frac{1}{2}C_{g}{V_{g}}^{2}+I_{d}\times t_{w}\times V$.

## 3 Conclusions

In conclusion, we demonstrate that a 10 nm Te_0.9_Se_0.1_ film deposited at low temperature exhibits a switchable electromechanical response and piezoelectric behavior. The Te_0.9_Se_0.1_ film preserves the crystal structure of Te while exhibiting enhanced non‐centrosymmetric properties, as evidenced by Raman spectroscopy and EXAFS analysis. PFM measurements reveal a measurable piezoresponse with hysteresis amplitude/phase, consistent with electrically tunable polarization‐related behavior. In addition, Te_0.9_Se_0.1_‐based memory exhibits quasi‐nonvolatile resistance modulation and reproducible program/erase characteristics, with endurance up to 10^3^ cycles and retention up to 2 × 10^3^ s. Finally, synaptic‐response measurements suggest the potential of Te_0.9_Se_0.1_ for neuromorphic signal sensing, achieving 92% image recognition accuracy and low‐energy operation under optimized device scaling. Overall, this work expands the piezoelectricity of Te─Se‐based p‐type thin films and provides a practical starting point for future improvements toward nonvolatile memory and neuromorphic electronic systems.

## 4 Experimental Session

### 4.1 Synthesis of Te_x_Se_1‐x_ Source

First, Te (Gredmann, 99.99%, powder) and Se (Gredmann, 99.99%, powder) were prepared with constant molar ratios equal to 9:1, 7:3, and 5:5, respectively. The powder was mixed and then ground homogeneously in a ball‐milling machine at 600 rpm for 3 h. Next, the ingot‐pressing machine was used to press the powder into ingots, thereby densifying the source.

### 4.2 Deposition of Te_x_Se_1‐x_ Films by Cryogenic Physical Vapor Deposition and Fabrication of Te_x_Se_1‐x_‐based Devices

First, the channel pattern was defined by photolithography with a channel width of 15 µm. Then, a continuous Te_x_Se_1‐x_ thin film was deposited on the 50 nm‐thick SiO_2_/P+Si substrate and 10 nm ZrO_2_/Al_2_O_3_/ZrO_2_/TiN/P+Si substrate by low‐temperature thermal evaporation. During deposition, the substrate temperature was set to −80°C using an ethanol cooling system, and the deposition rate was controlled at 1 Å/s with a chamber pressure below 4 × 10^−7 ^Torr. Next, the temperature was set to 0°C after the deposition to crystallize the film for 1 h. After channel deposition, the second photolithography process was used to define the source/drain pattern with a channel length of 13 µm. Next, 50 nm Au was deposited on the substrate by thermal evaporation at room temperature.

### 4.3 Measurements of Piezoelectric Force Microscopy (PFM)

The samples for vertical PFM measurements were fabricated by depositing Te_x_Se_1‐x_ thin films onto an Au‐coated P+Si substrate. An AC bias voltage (V_AC_) was used to drive the PFM tip, and its response (including both amplitude and phase lag from the drive) was recorded. When different V_AC_ voltages (0.5, 1, 1.5, 2, 2.5) are applied, the average amplitudes increase linearly if the materials exhibit piezoelectric effects. The slope of the increase of amplitude changes with V_AC_ to get enough signal is the piezoelectric constant (d_33_). For domain writing/poling, the conductive tip was scanned in contact mode while applying a DC bias (V_DC_ = ± 5 V) with V_AC_ = 500 mV (on‐field condition). The applied electric field can induce polarization switching of piezoelectric domains. After removal of V_DC_, the remanent domain contrast was examined by PFM amplitude and phase mapping (off‐field imaging). To evaluate hysteresis behavior, switching spectroscopy PFM (SS‐PFM) measurements were performed. In SS‐PFM, V_DC_ was swept between +5 V and −5 V (instead of applying only a constant ± 5 V) at a scanning frequency of 1 Hz, while V_AC_ = 500 mV was maintained. Piezoresponse signals were collected to construct both on‐field and off‐field phase‐voltage and amplitude‐voltage hysteresis loops. In particular, the off‐field SS‐PFM readout (recorded after removal of the DC bias at each bias step) was used to reduce electrostatic and charge‐injection contributions when evaluating polarization switching behavior.

### 4.4 FET and Memory Measurements

The FET characteristics of the Te_x_Se_1‐x_‐based FET were measured using a semiconductor parameter analyzer (Agilent B1500A). Pulsed memory tests of the devices were performed using a semiconductor parameter analyzer (Tektronix, Keithley 4200‐SCS). A semiconductor parameter analyzer (Keithley 4200) coupled with a pulse generator unit (Tektronix, Keithley 4220‐PGU) and an oscilloscope (Tektronix, Keithley 4200‐SCP2) with 2 channels was used.

### 4.5 Material Characterization

The bonding characteristics of different ratios of Te_x_Se_1‐x_ were examined by Raman spectroscopy using a UniDRON confocal microscope (Raman/PL Spectroscopy System) with DPSS laser excitation (532 nm, 100 mW) at an applied power of 10 mW, using a 50× objective and a high‐sensitivity TE‐cooled charge‐coupled device detector. The crystal structures were examined by X‐ray diffraction (XRD, Bruker, D8 Discover X‐ray Diffraction System). And the d‐spacing values in each plane is calculated from Bragg's Law $d=\frac{n\lambda }{2\sin\theta }$, for whichλis 0.1541 nm (Cu Kα). The atomic structure of plain‐view Te_x_Se_1‐x_ thin film was characterized using a high‐resolution transmission electron microscope (HRTEM, JEM‐F200, JEOL) and (ARM ‐200FTH) equipped with an energy dispersive X‐ray spectroscope (EDS). The crystal grain size and structure space group were defined by Electron backscattered Diffraction (EBSD, Zeiss Gemini 300 Scanning Electron Microscope). The Extended X‐Ray Absorption Fine Structure (EXAFS) was measured in NSRRC TPS‐44A. The piezoelectric constant and polarization domain were examined by Piezoelectric Force Microscopy (PFM, Asylum Research, Cypher S AFM Microscope) on the gold substrate. A conductive Pt–Ir coated cantilever (PPP‐NCSTPt‐10, Nanosensors) with a force constant of around 7.4 N m^−1^ and a contact resonance frequency of around 160 kHz was applied. Local piezoresponse hysteresis loops were obtained via spectroscopic piezoresponse force microscopy (SS‐PFM) using a triangular voltage waveform.

## Author Contributions

C.C.C. and Y.L.C. conceived and coordinated the study. C.C.C. performed the experiments, analyzed the data, and constructed the curves and figures. M.C. measured the neural synaptic results. C.H.L., P.C.L., Y.J.L., R.H.C., Y.R.P., B.N.G., Z.F.L., Q. T. L., and Y.J.Y. helped in the experiments and materials analysis. C.C.C. and Y.L.C. wrote the manuscript with contributions from all the co‐authors. Y.F.L., M.H.L., Y.H.C., C.H.S., D.H.L., and Y.L.C. provided theoretical guidance. All authors discussed the results and commented on the manuscript.

## Conflicts of Interest

The authors declare no conflicts of interest.

## Supporting information




**Supporting File**: advs75407‐sup‐0001‐SuppMat.docx.

## Data Availability

The data that support the findings of this study are available in the supplementary material of this article.
