# Supplementary material for: Polarization‐Enabled Piezoelectric Tellurium–Selenium (Te x Se1– x ) Thin Films for Memory Switching and Artificial Synaptic Functions
Source: Adv Sci (Weinh). 2026 May 27;13(40):e24308. doi: 10.1002/advs.202524308 (PMC13335475; doi:10.1002/advs.202524308)
Supplement: Supplementary file 1 — Supporting File: advs75407‐sup‐0001‐SuppMat.docx. [file ADVS-13-e24308-s001.docx]

**Supporting Information**

**Polarization-Enabled Piezoelectric Tellurium–Selenium (Te*_x_*Se_1–_*_x_*) Thin Films for Memory Switching and Artificial Synaptic Functions**

Chia-Chen Chung^1^, Mayur Chaudhary^1^, Chia-Hung Lo^1^, Po-Chien Lai^1^, You-Jie Lin^1^, Ruei-Hong Cyu^1, 4^, Yu-Ren Peng^1, 4^, Bing-Ni Gu^1^, Zhao-Feng Lou^8^, Quynh Thi Le^1^, Yi-Jen Yu^5^, Yen-Fu Lin^9^, Min-Hung Lee^8^, Ying-Hao Chu^1^, Chang-Hong Shen^2^, Der-Hsien Lien^*6^, Yu-Lun Chueh^1, 2, 3, 7*^

^1^Department of Materials Science and Engineering, National Tsing Hua University, Hsinchu 30013, Taiwan

^2^College of Semiconductor Research, National Tsing-Hua University, Hsinchu, 30013, Taiwan

^3^Department of Physics, National Sun Yat-Sen University, Kaohsiung, 80424, Taiwan

^4^Ph. D. Program in Prospective Functional Materials Industry, National Tsing Hua University, Hsinchu 30013,

Taiwan
^5^Instrument Center, National Tsing Hua University, Hsinchu, 30013, Taiwan

^6^Department of Electronics Engineering, National Yang Ming Chiao Tung University, Hsinchu 30013, Taiwan

^7^Department of Materials Science and Engineering, Korea University, Seoul 02841, Republic of Korea

^8^Graduate School of Advanced Technology, National Taiwan University, Taipei 106319, Taiwan

^9^Department of Physics, National Chung Hsing University, Taichung, 40227, Taiwan

*E-mail:ylchueh@mx.nthu.edu.tw and dhlien@nycu.edu.tw

**Table S1.** d-spacing value for Te_x_Se_1-x_ thin films with different compositions calculated by Bragg’s Law.

| **Materials** | **(100) (Å)** | **(101) (Å)** | **(110) (Å)** | **(111) (Å)** |
| --- | --- | --- | --- | --- |
| Te_0.9_Se_0.1_ | 3.833 | 3.191 | 2.219 | 2.069 |
| Te_0.7_Se_0.3_ | 3.817 | 3.131 | 2.208 | 2.047 |
| Te_0.5_Se_0.5_ | 3.801 | 3.094 | 2.203 | 2.032 |

**
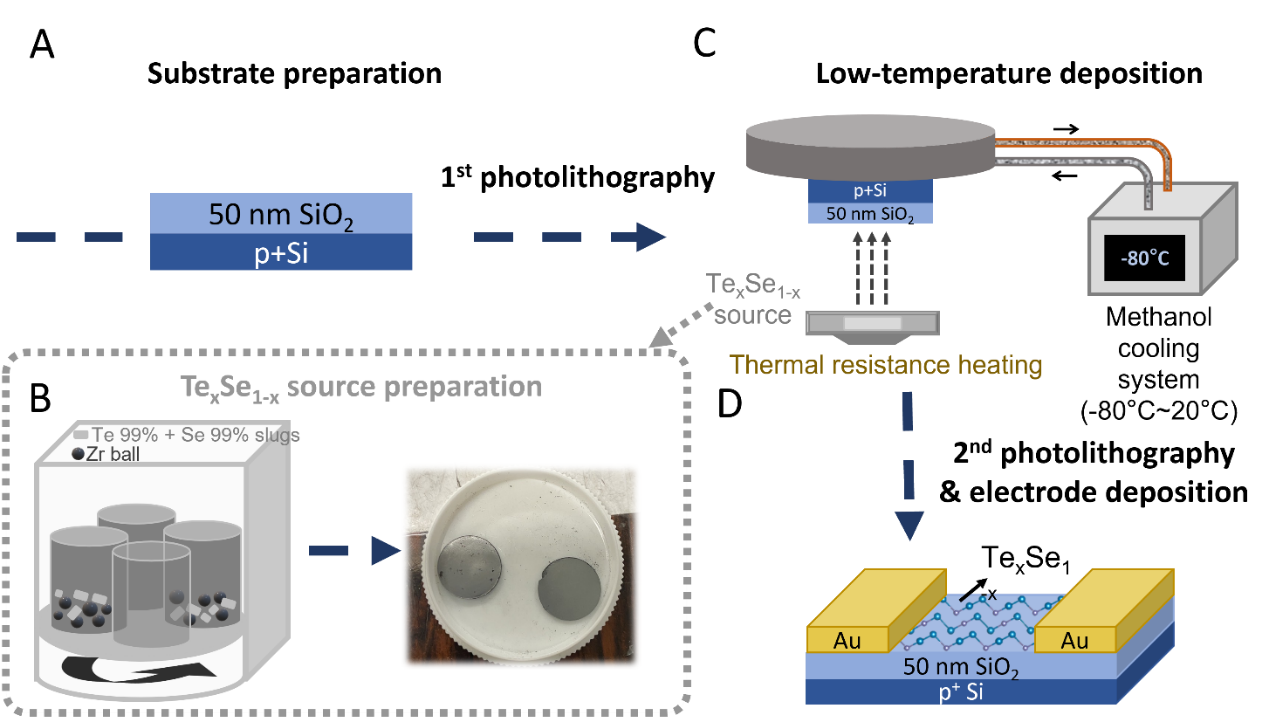
**

**Figure S1** Schematic process flow of Te_x_Se_1-x_ thin film-based devices and Te_x_Se_1-x_ source preparation


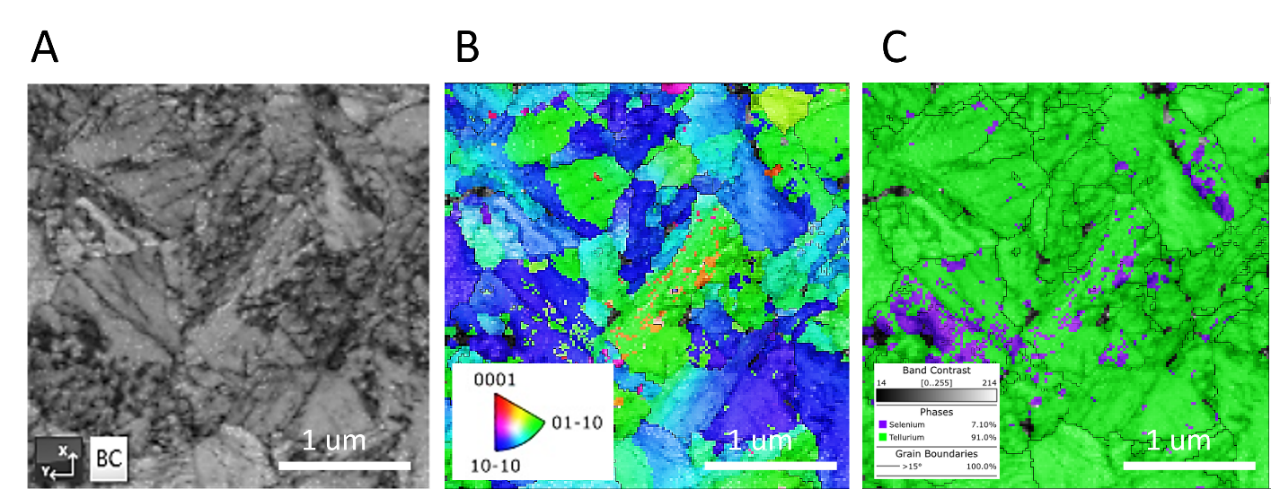


**Figure S2 EBSD mapping images of the plane-view of 10 nm-thick Te_0.9_Se_0.1_ thin film** (A) SEM band contrast image (B) Inverse Pole Figure (IPF) z-axis image, which corresponds to space group P3_1_21 (152) Tellurium (C) Phase diagram of the ratio of Tellurium and Selenium

**
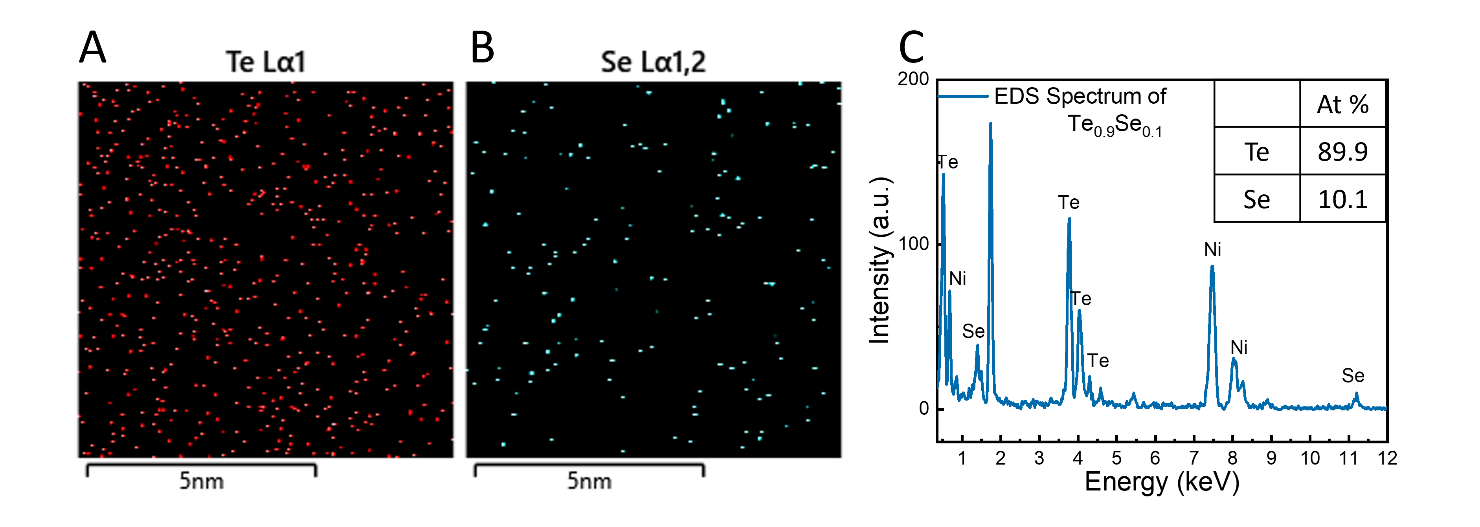
**

**Figure S3 Plane-view EDS mapping results of 10 nm-thick Te_0.9_Se_0.1_ thin film** (A) Tellurium Lα 1 spectrum of the film(B) Selenium Lα 1 spectrum of the film (C) Entire element spectrum of Te, Se and Ni. The signal of Ni is due to that the film is deposited on the Ni grid.

**
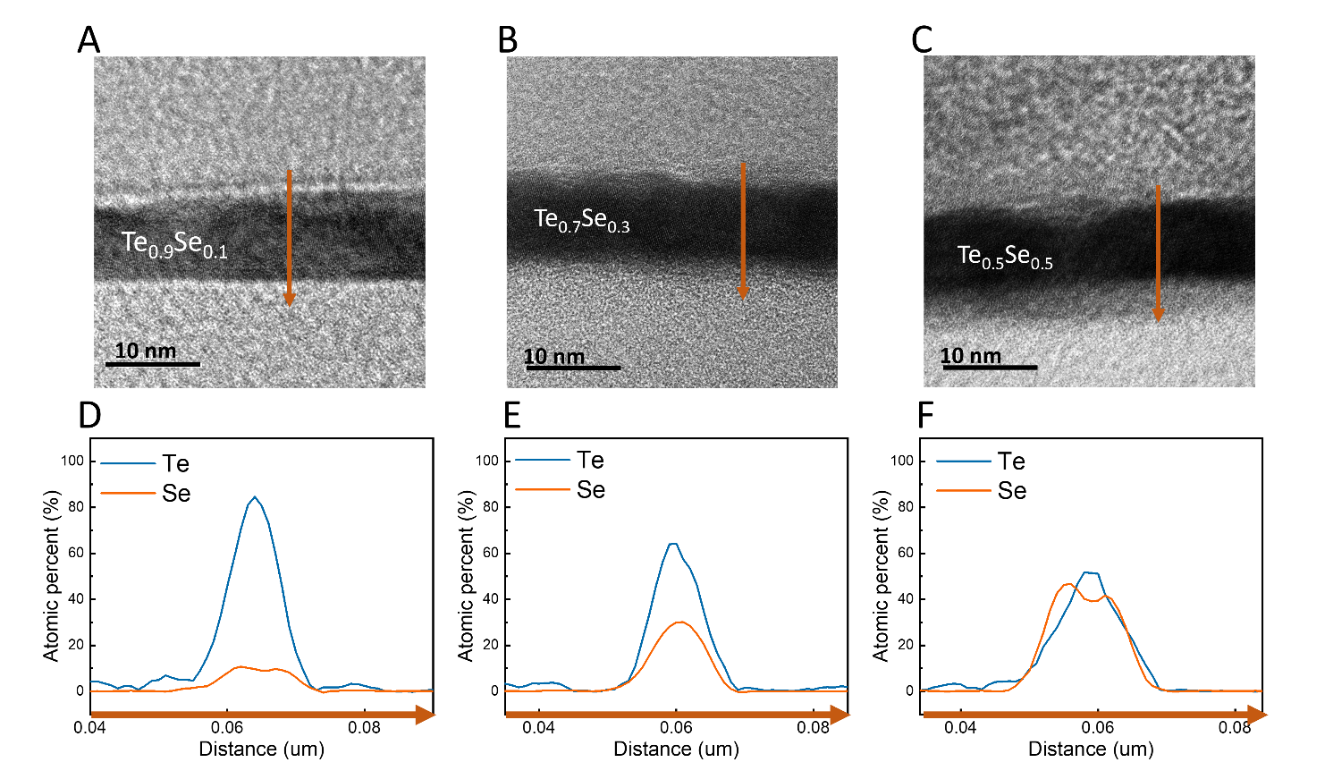
**

**Figure S4 STEM-EDS Line scanning of cross-sectional Te_x_Se_1-x_ thin film-based devices** (A,D) Te_0.9_Se_0.1_; (B,E) Te_0.7_Se_0.3_; (C,F) Te_0.5_Se_0.5_.

**
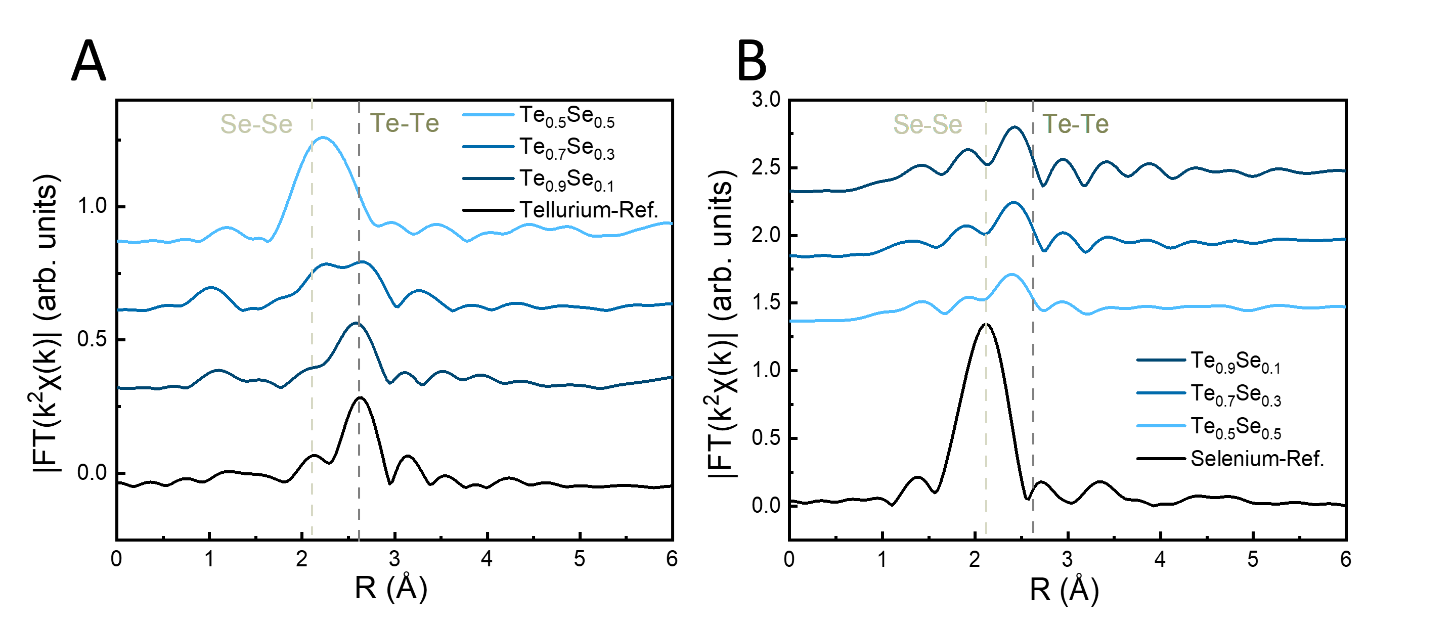
**

**Figure S5 Corresponding Fourier transform of k^3^-weighted EXAFS spectrum.** (A) Te K-edge spectrum with different Tellurium ratios. (B) Se K-edge spectrum with different Selenium ratios.

**
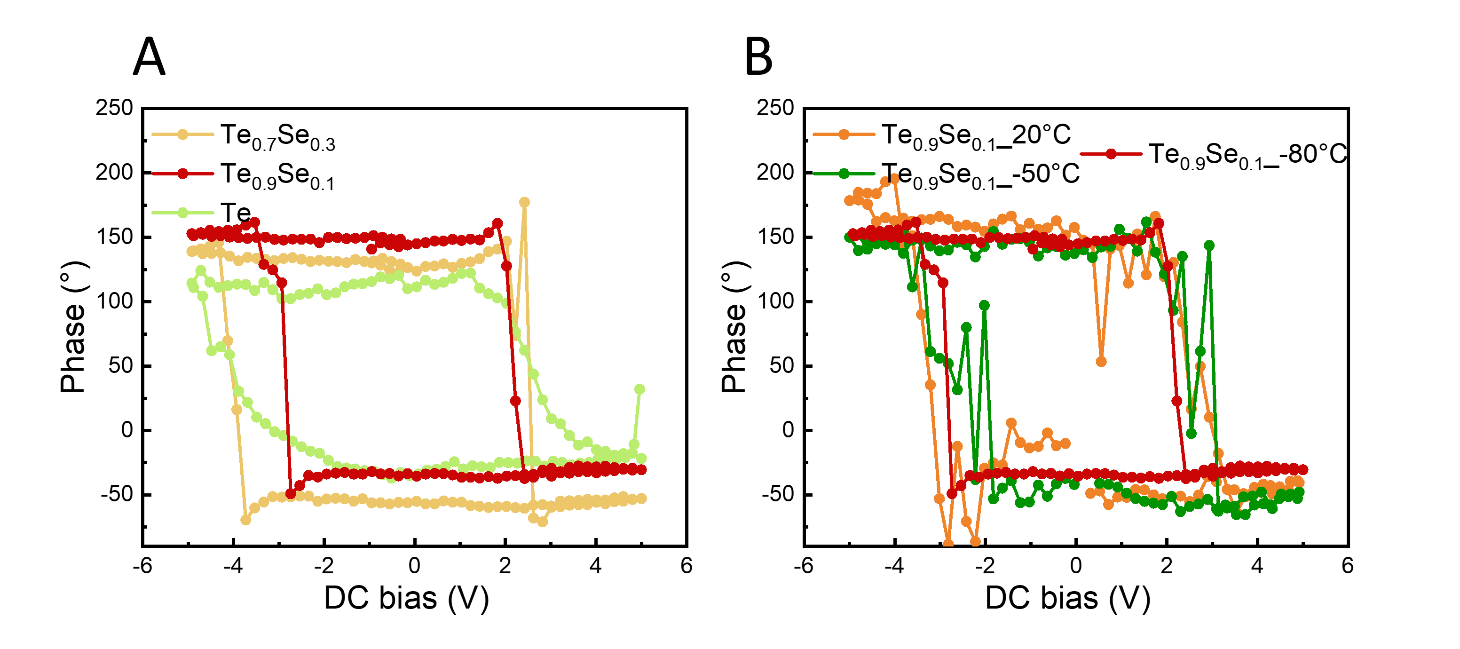
**

**Figure S6** **Off-field phase–voltage hysteresis loops of different ratios Te_x_Se_1-x_ and deposition temperatures_._** (A) 10 nm-thick pure Tellurium, Te_0.9_Se_0.1_ and Te_0.7_Se_0.3_; (B) 10 nm Te_0.9_Se_0.1_ deposited at -80°C, -50°C and 20°C

**
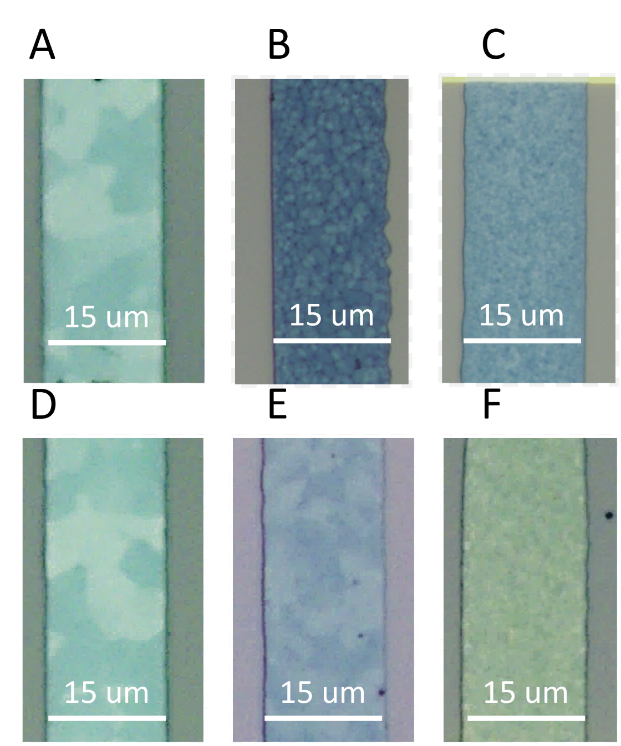
**

**Figure S7** **OM images of 10 nm Te_x_Se_1-x_ with different ratios and deposition temperatures.** Te_0.9_Se_0.1_ at the deposited temperatures of (A) -80°C, (B) -50°C, (C) 20°C, (D) Te_0.9_Se_0.1_, (E) Te_0.7_Se_0.3_, and (F) Te_0.5_Se_0.5_ at the fixed deposited temperature of -80°C.

**
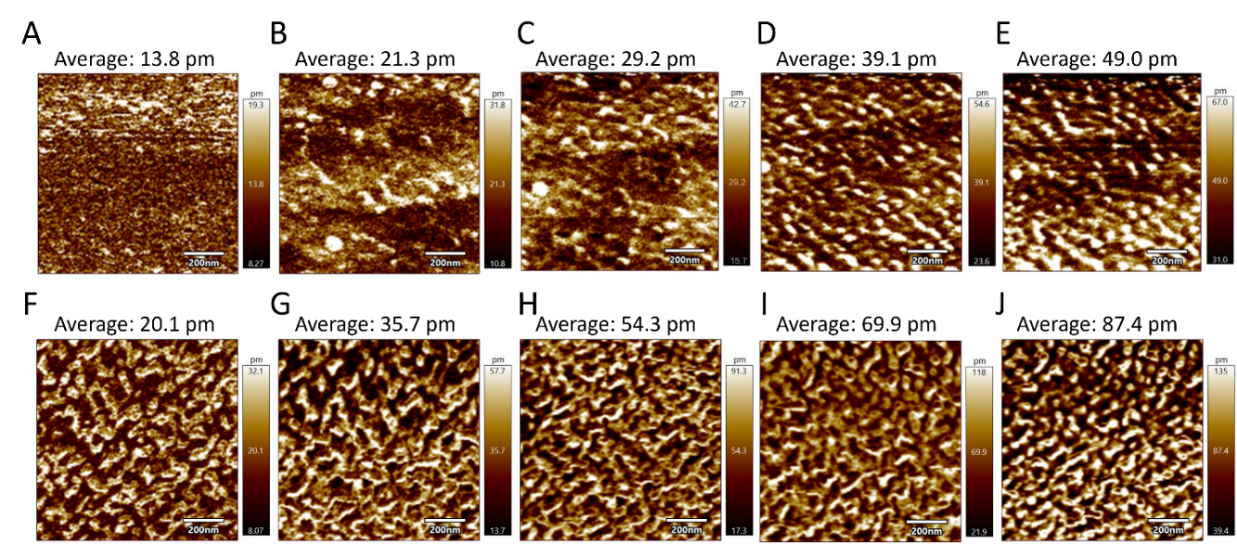
**

**Figure S8 Average amplitude variation of 10 nm-thick Te and Te_0.9_Se_0.1_ thin films with different V_AC_** (A) V_AC_= 0.5 V, (B) V_AC_ = 1 V, (C) V_AC_ = 1.5 V, (D) V_AC_ = 2 V, (E) V_AC_ = 2.5 V. Average amplitude variation of 10 nm Te_0.9_Se_0.1_ with different V_AC_ (F) V_AC_ = 0.5 V, (G) V_AC_ = 1 V, (H) V_AC_= 1.5 V, (I) V_AC_= 2 V, (J) V_AC_ = 2.5 V.

**
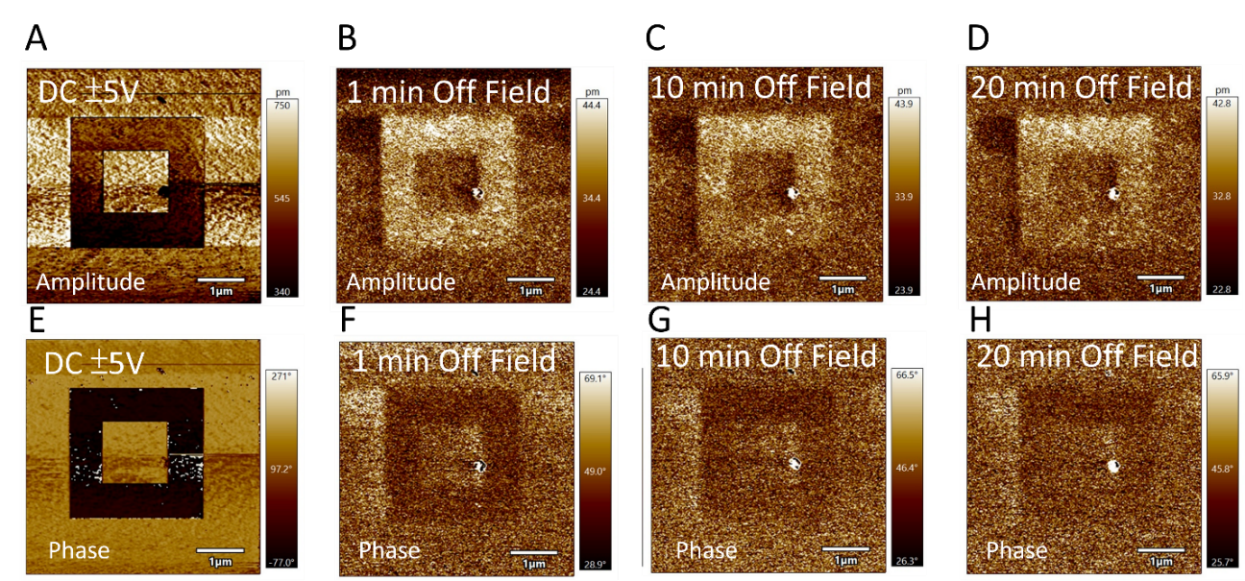
**

**Figure S9 Mapping of residue of domain.** (A,E) Response of PFM amplitude and phase of Te_0.9_Se_0.1_ thin film with V_DC_ = ± 5V. (B,F) Response of PFM amplitude and phase 1 minute after removing V_DC_. (C,G) Response of PFM amplitude and phase 10 minutes after removing V_DC_. (D,H) Response of PFM amplitude and phase 20 minutes after removing V_DC_.

**
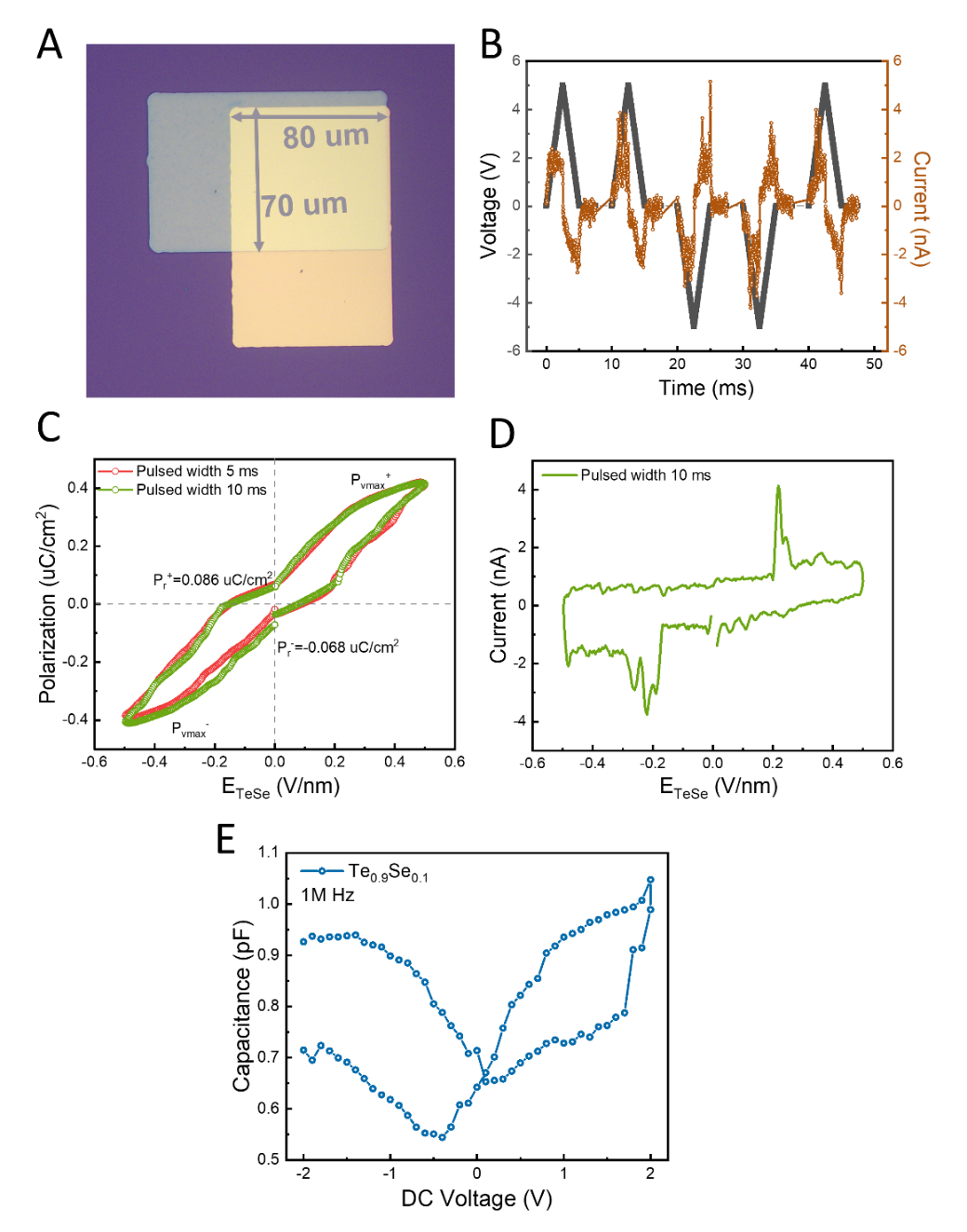
**

**Figure S10 Polarization switching and dielectric response of Au/Te_0.9_Se_0.1_** **/Au capacitors.** (A) OM image of the crossbar capacitor structure used for PUND and C–V measurements, showing the overlap area (≈ 80 × 70 µm^2^). The thickness of Te_0.9_Se_0.1_ is 10 nm. (B)Schematic of the triangular input pulse sequence (pulse width: 5 ms) and the corresponding pulse-resolved I–t transient curve. (C) PUND-extracted polarization–electric field (P–E) hysteresis loops measured at voltage ranges equal to ±0.5 V/nm, with pulsed width 5 ms and 10 ms. The measured remanent polarization values are P_r_^+^= 0.086 μC/cm^2^ and P_r_^-^= -0.068 μC/cm^2^, respectively. (D) PUND-extracted current–electric field (I–E) hysteresis loops. (E) Capacitance–voltage (C–V) characteristics of the same Au/Te_0.9_Se_0.1_/Au capacitor measured at 1 MHz, showing a butterfly-like enhancement in capacitance near the switching-voltage region.


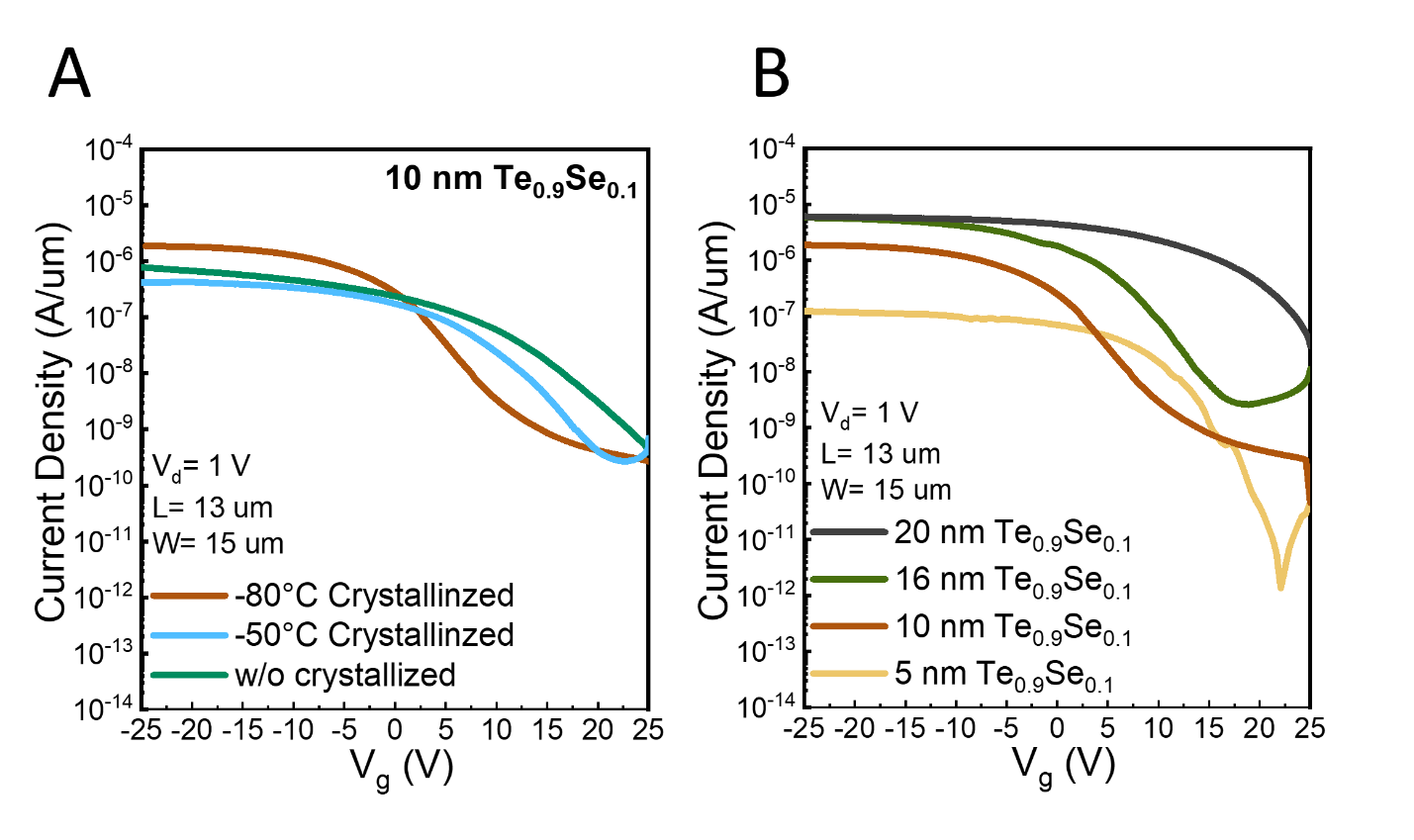


**Figure S11 Transistor behaviors of different deposited temperatures and thicknesses.** (A) I_d_-V_g_ transfer curves of Te_0.9_Se_0.1_ thin film-based FETs with different deposited temperatures. (B) I_d_-V_g_ transfer curves of Te_0.9_Se_0.1_ thin film-based FETs with different thicknesses.

**Supporting note 1:**

To examine whether trap-assisted charging/discharging contributes to the observed hysteresis, transfer characteristics were measured at different gate sweeping rates, as shown in Figure S12A. The inset summarizes the dependence of hysteresis width as the function of the sweeping rate. Consistent with prior reports on 2D FETs, trap-assisted charging/discharging is generally a relatively slow process; therefore, a slower gate sweep, corresponding to a longer dwell time, can lead to a larger apparent hysteresis because more carriers have sufficient time to be trapped and de-trapped during the scan^[1]^. In our measurements, the hysteresis indeed becomes larger at the slower sweeping rate of 0.55 V/s. Meanwhile, a clear hysteresis is still retained when the sweep rate is increased to 10 V/s. These results suggest that trap-assisted processes may contribute to the hysteresis, but they are unlikely to fully account for the observed behavior within the tested sweep-rate window. We therefore present the sweep-rate study as supporting evidence rather than as a comprehensive kinetic separation of the underlying mechanism. Moreover, we annealed the 10 nm Te_0.9_Se_0.1_-based FETs at 140 °C and compared the transfer hysteresis before and after annealing (Figure S12B). Post-annealing may partially reduce trap densities and improve contact quality. Notably, both devices exhibit a clockwise hysteresis, while the annealed sample shows a larger hysteresis behavior, suggesting that the observed switching is not suppressed by trap reduction and instead becomes more pronounced when defect-related interference is alleviated. In addition, stoichiometric TeO_2_ is generally regarded as non-ferroelectric crystal class^[2]^. We therefore used TeO_2_ as a reference device (Figure S12C), which exhibits counterclockwise hysteresis that is commonly associated with trapping-dominated behavior. The TeO_2_ device shows a counterclockwise hysteresis, consistent with trapping-dominated behavior rather than polarization-like switching. Taken together, the persistence of the clockwise hysteresis under fast sweeps and after trap-mitigating annealing, combined with the opposite (counterclockwise) hysteresis observed in the TeO_2_ control, suggests that charge trapping is unlikely to be the sole origin of the hysteresis direction in Te_0.9_Se_0.1_ devices, and is consistent with a polarization-related contribution. Although this sweep-rate study is not intended as a complete kinetic separation across all possible time scales, it nevertheless provides useful evidence to partially differentiate trapping-related effects from the observed hysteresis behavior.

Moreover, based on the PUND measurement, the remanent polarization is on the order of P_r_ ≈ 0.08 μC cm^−2^. Using the effective capacitor area A = 0.0056 mm^2^ = 5.6×10^−5^ cm^2^, the corresponding switched charge can be estimated as Q ≈ P_r_ × A ≈ (0.08×10^−6^) C cm^−2^ × (5.6×10^−5^) cm^2^ ≈ 4.48×10^−12^ C. Since the capacitance of the actual back-gated transistor channel cannot be directly isolated because of substantial parasitic contributions, the capacitance measurement was performed on a separate Au/Te_0.9_Se_0.1_/Au overlap capacitor structure to evaluate the film-related response. With the measured capacitance of the overlap structure C ≈ 0.6 pF, the polarization-induced gate-equivalent electrostatic modulation is therefore ΔV ≈ Q/C ≈ 7.46 V. Therefore, the present ΔV analysis is intended only as a rough consistency check, rather than as a strict quantitative closure, showing that the voltage scale estimated from the measured polarization is comparable to the experimentally observed transfer hysteresis width (effective V_th_ shift) of ∼8 V in Figure S12A.


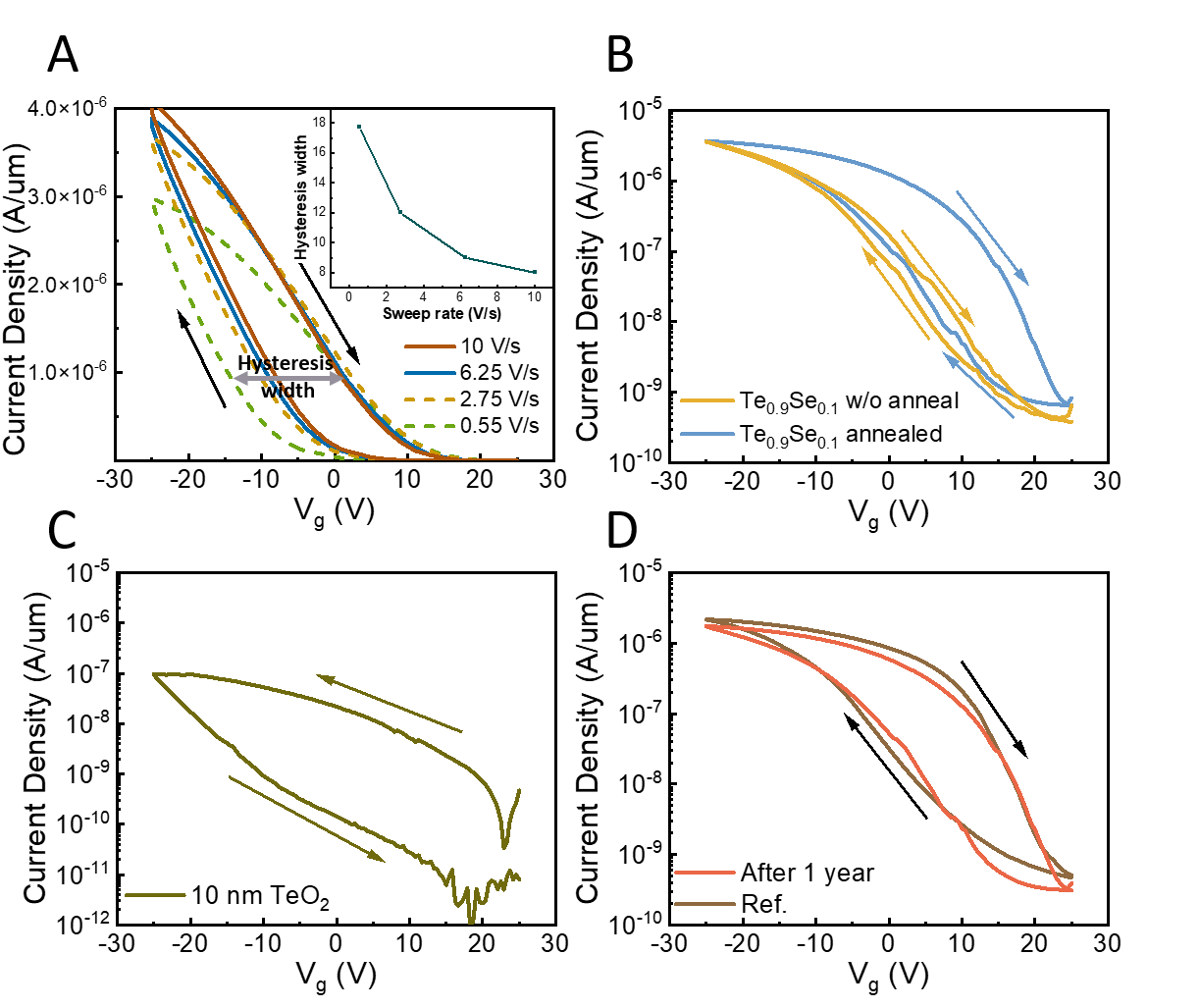


**Figure S12** **Influence of trap effect and stability performance** (A) Hysteresis transfer characteristics of Te_0.9_Se_0.1_ thin film-based FET with different sweeping rates ranging from 0.55V/s to 10 V/s under V_d_= 1V. The inserted figure is the trend of hysteresis width varied with sweeping rate. (B) Hysteresis transfer characteristics of Te_0.9_Se_0.1_ thin-film FETs under V_d_= 1V measured with and without post-annealing, showing the corresponding hysteresis behavior. (C) Hysteresis transfer characteristics of a 10 nm TeO_2_-based FET under V_d_= 1V. (D) Hysteresis transfer characteristics of Te_0.9_Se_0.1_ thin film-based FET under V_d_= 1V before and after one year.


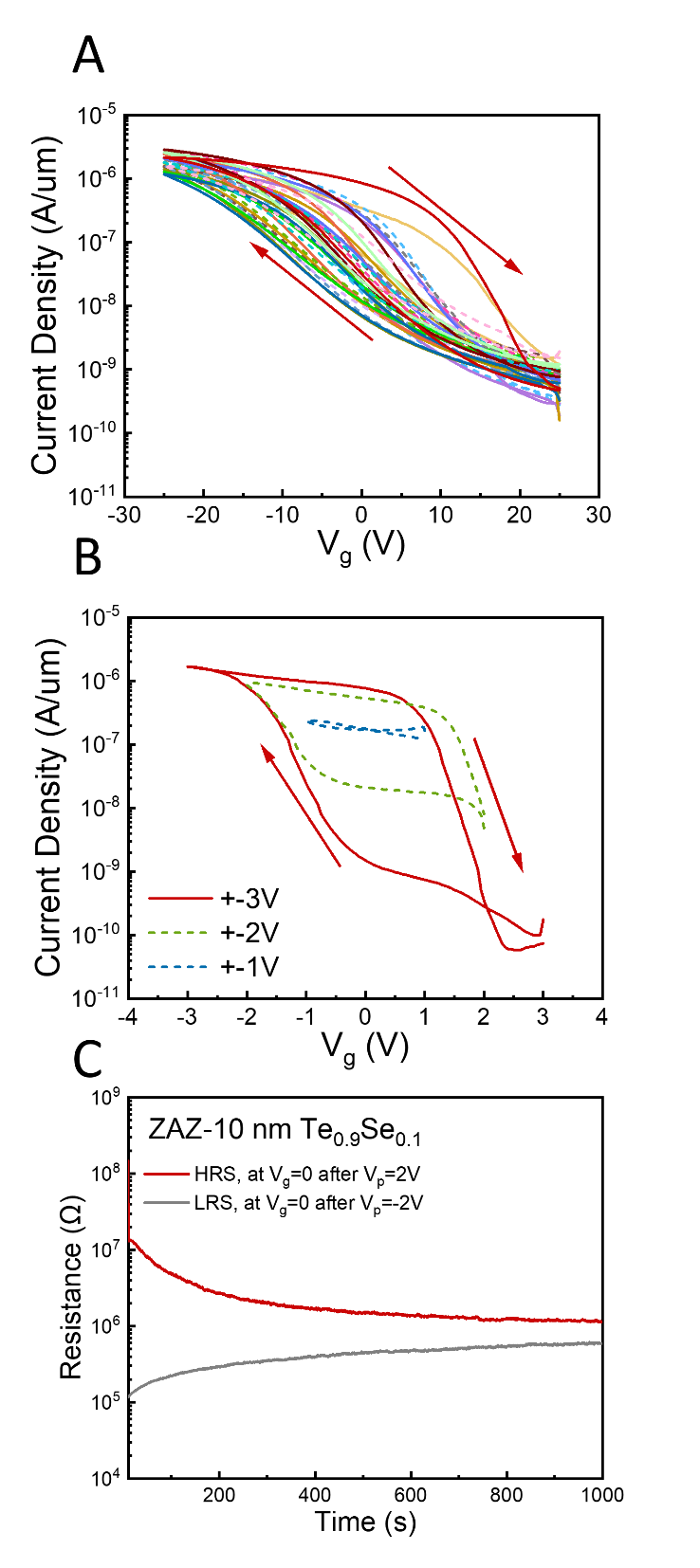


**Figure S13 Repeatability and substrate characteristics.** (A) Hysteresis transfer characteristics of 20 Te_0.9_Se_0.1_ thin film-based FET devices (B) Hysteresis transfer characteristics of Te_0.9_Se_0.1_ thin film-based FET with ZrO_2_/Al_2_O_3_/ZrO_2_ (ZAZ) as gate dielectric layer, gate voltage ranging from 3V~-3V, 2V~-2V and 1V~-1V. The drain voltage is set as 1 V and the direction of hysteresis loops shows clockwise. (C) Quasi-nonvolatile memory retention characteristics of Te_0.9_Se_0.1_ with ZAZ as gate dielectric layer, with program/ease pulsed voltage of ±2 V and a pulse width of 10 ms, followed by read-after-delay at V_g_ = 0 V and V_d_ = 1 V.


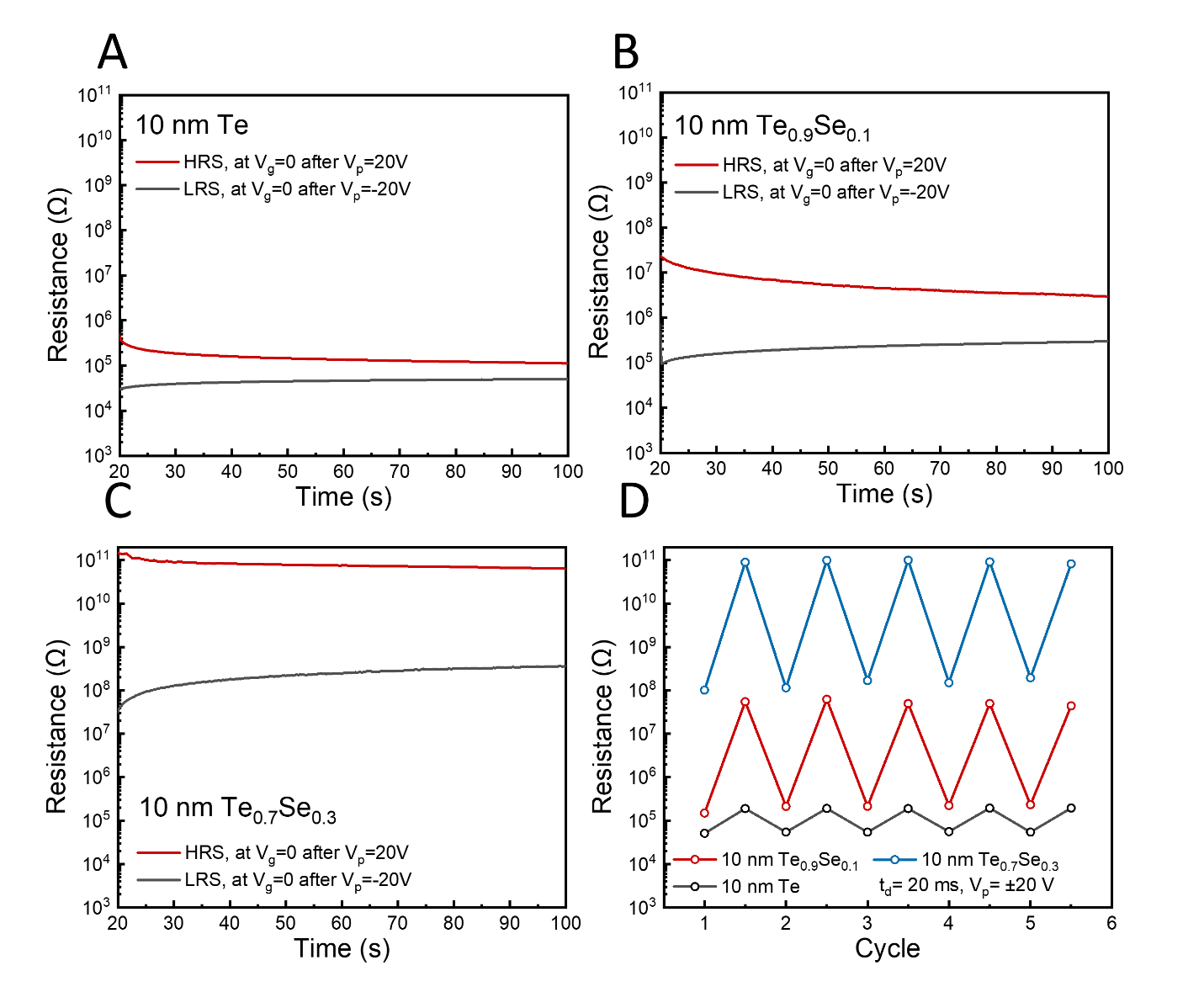
**Figure S14 Quasi-nonvolatile memory retention characteristics of different ratios Te_x_Se_1-x_ with** **program/ease pulsed voltage** **of ±20 V and a pulse width of 10 ms, followed by read-after-delay at V_g_ = 0 V and V_d_ = 1 V** (A) 10 nm Tellurium (B) 10 nm Te_0.9_Se_0.1_ (C) 10 nm Te_0.7_Se_0.3_. (D) The cycle test of different ratios Te_x_Se_1-x_ thin film-based memory devices.


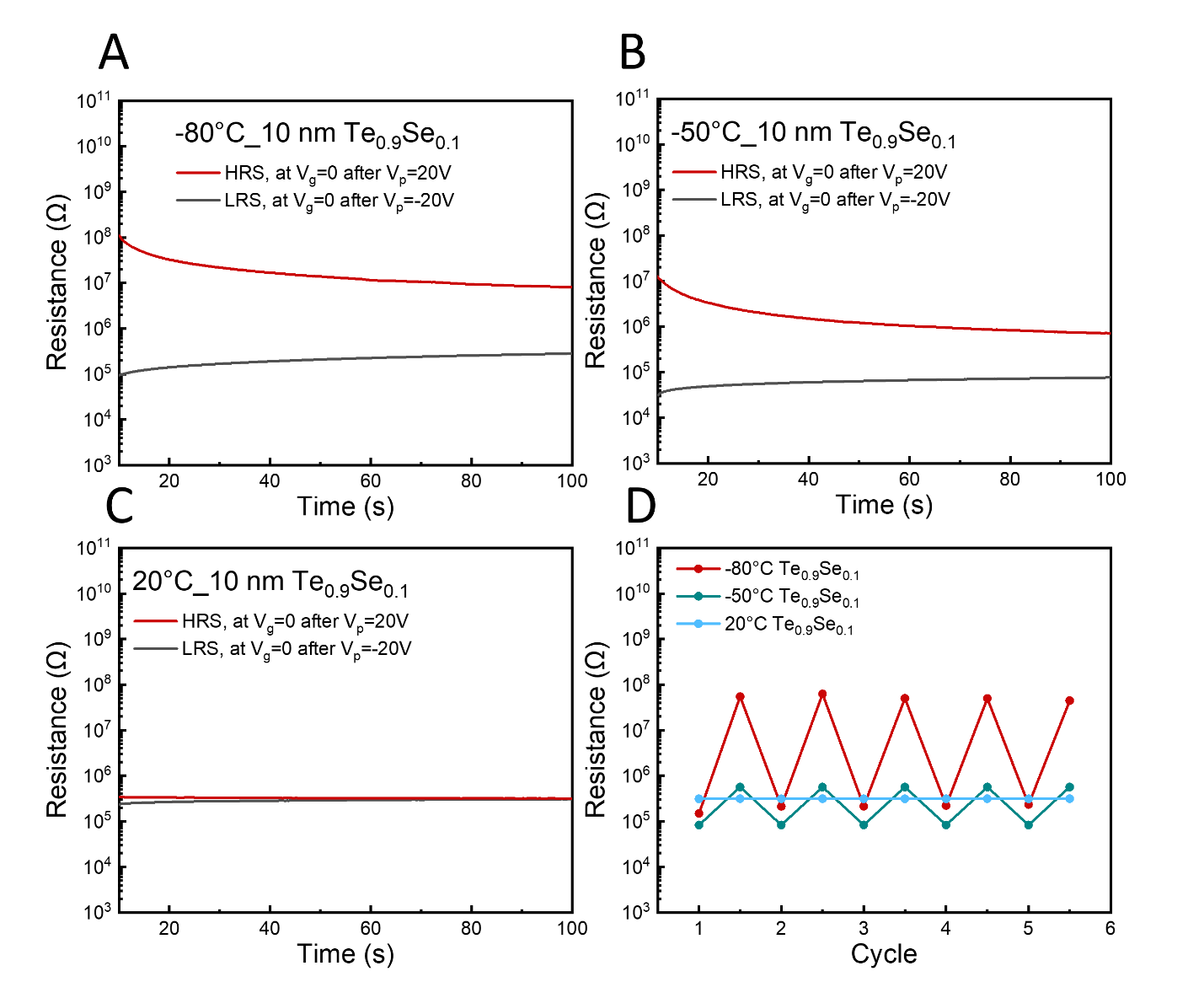


**Figure S15 Quasi-nonvolatile memory retention characteristics of different deposition temperatures of Te_0.9_Se_0.1_ with of ±20 V and a pulse width of 10 ms, followed by read-after-delay at V_g_ = 0 V and V_d_ = 1 V** (A) -80°C, (B) -50°C, (C) 20°C. (D) The cycle test of different deposition temperature Te_x_Se_1-x_ thin film-based memory devices.


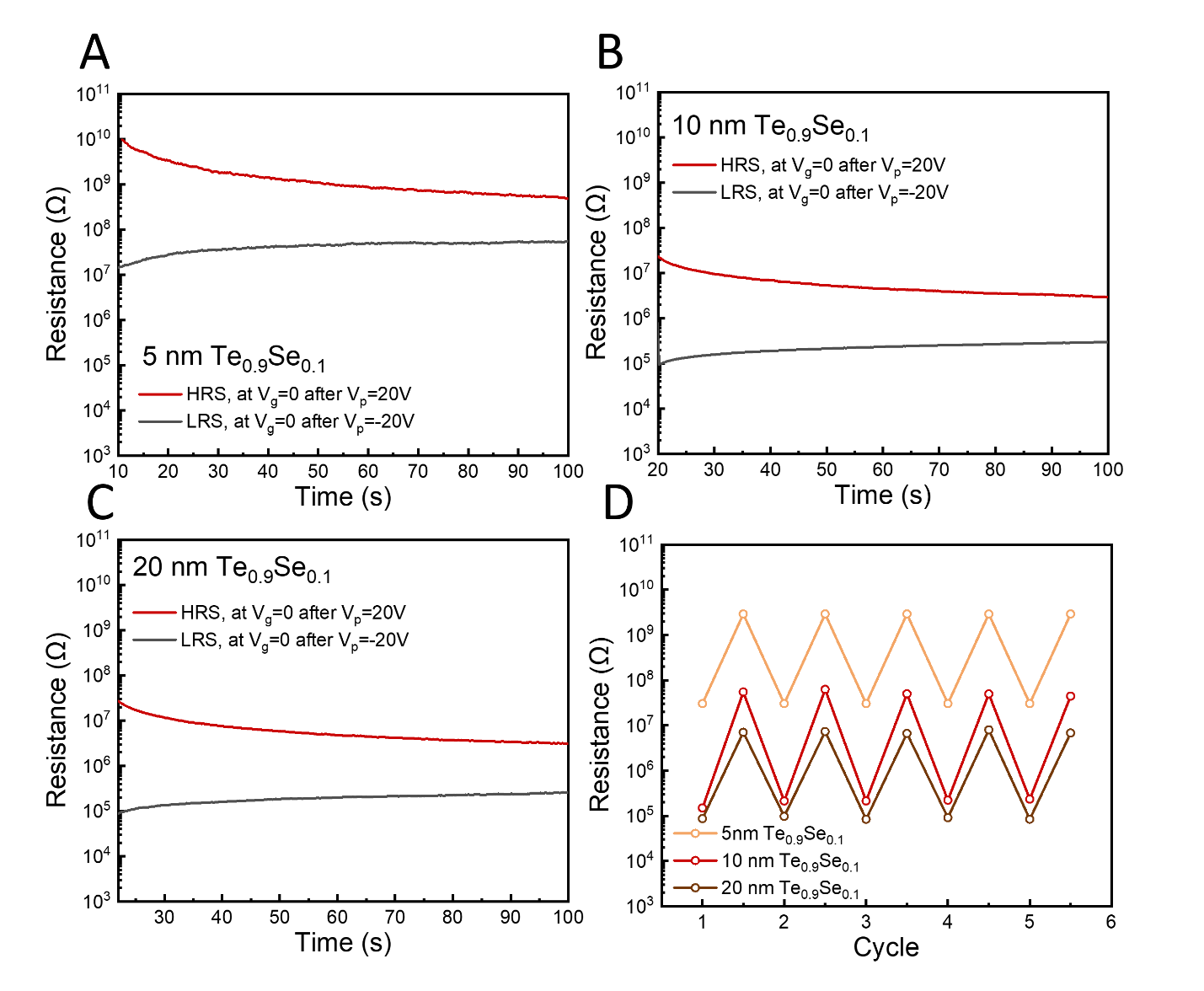


**Figure S16 Quasi-nonvolatile memory retention characteristics of different thicknesses Te_0.9_Se_0.1_ with of ±20 V and a pulse width of 10 ms, followed by read-after-delay at V_g_ = 0 V and V_d_ = 1 V** (A) 5 nm (B) 10 nm (C) 20 nm. (D) The cycle test of different thicknesses Te_0.9_Se_0.1_ thin film-based memory devices.


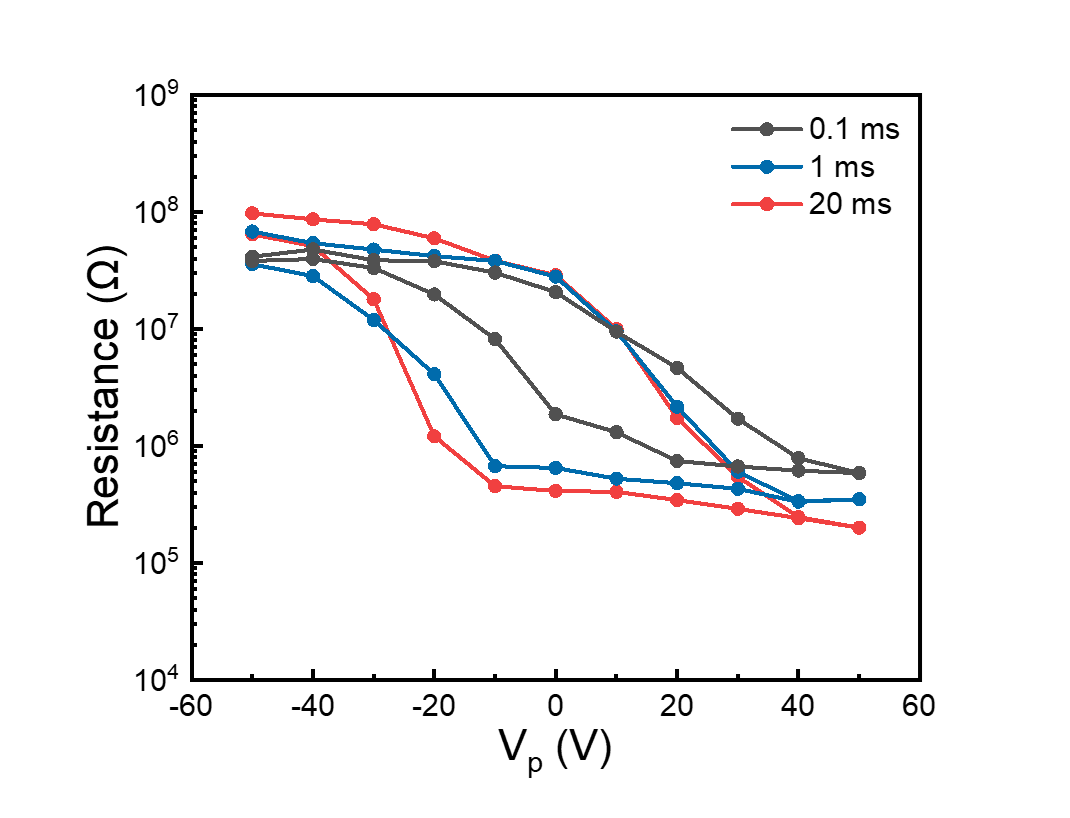


**Figure S17 Hysteresis loop of resistance versus pulsed voltage.** The pulsed widths are 0.1 ms, 1 ms, and 20 ms, respectively. Measurement of each data point is performed at *V*_g_ = 0 V after waiting for 1 min following applying each *V*_p_ pulse.

**
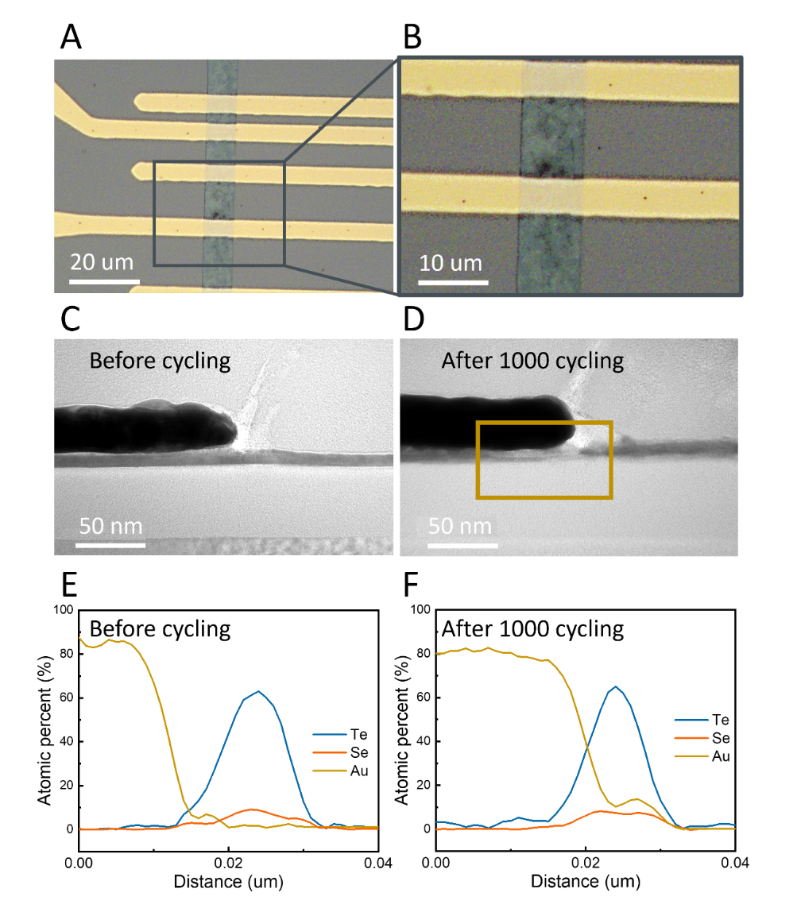
**

**Figure S18 Analysis of the memory device after 1000 cycling tests.** (A) OM image of the Te_0.9_Se_0.1_ memory device after 1000 program/erase cycles. (B) Magnified OM image highlighting the degraded/failure region. (C,D) Cross-sectional TEM images of the device (C) before cycling and (D) after 1000 cycles. (E,F) Corresponding EDS line-scan profiles (E) before cycling and (F) after 1000 cycles.


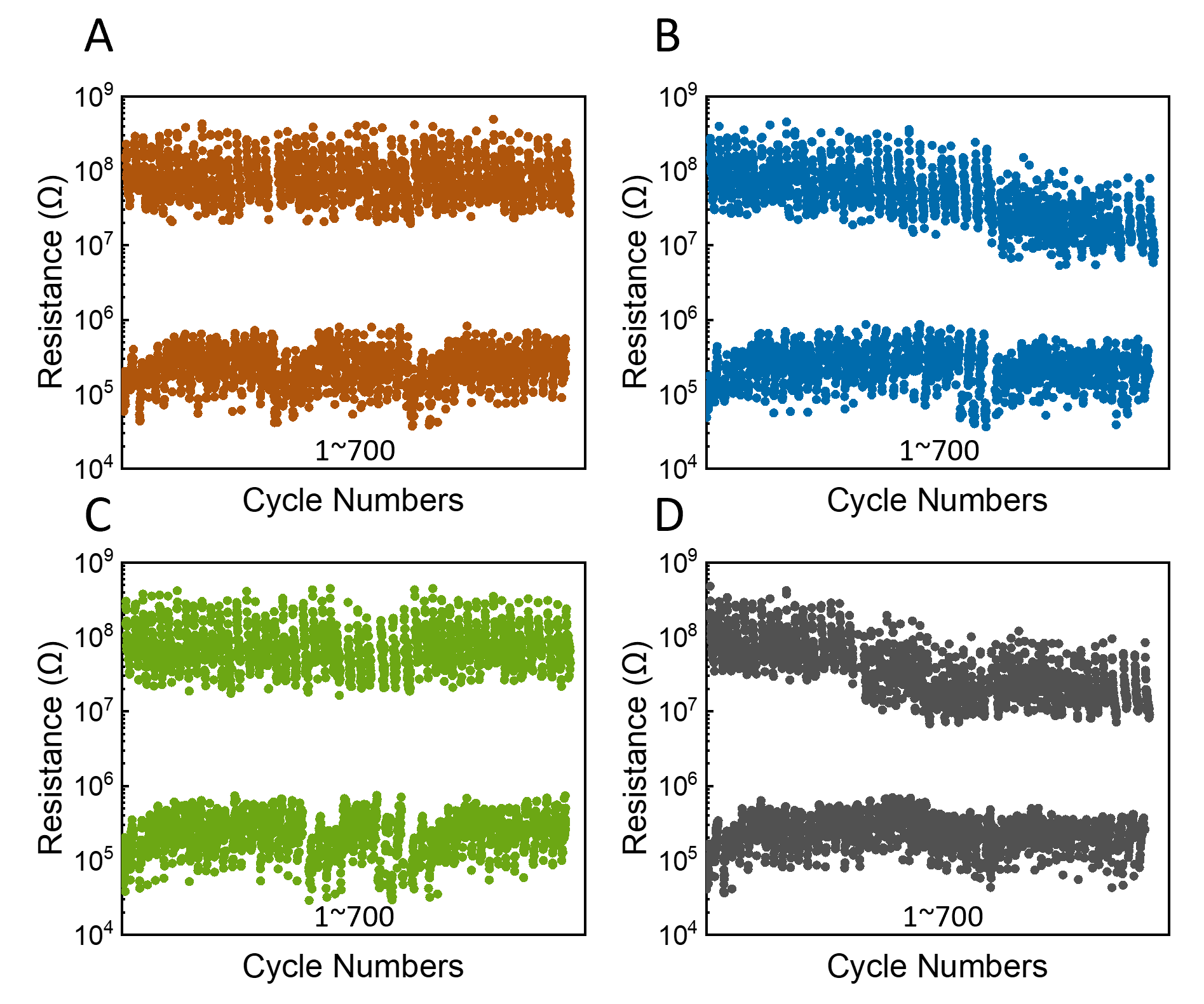

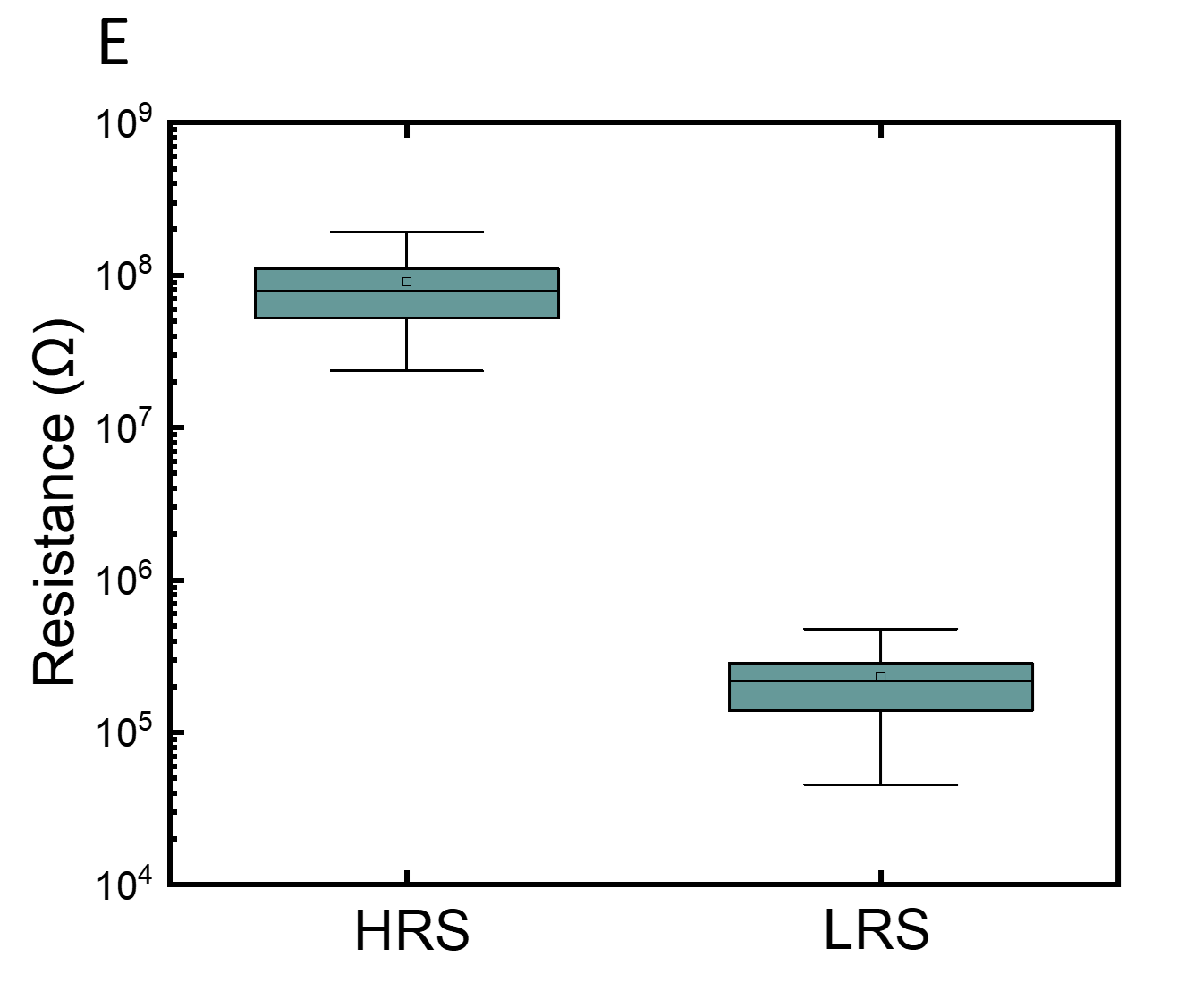


**Figure S19 Multi-device endurance test.** (A-D) Endurance characteristics of different Te_0.9_Se_0.1_ thin-film–based memory devices measured 700 program/erase (write/erase) cycles. (E) Error bar of the HRS and LRS.


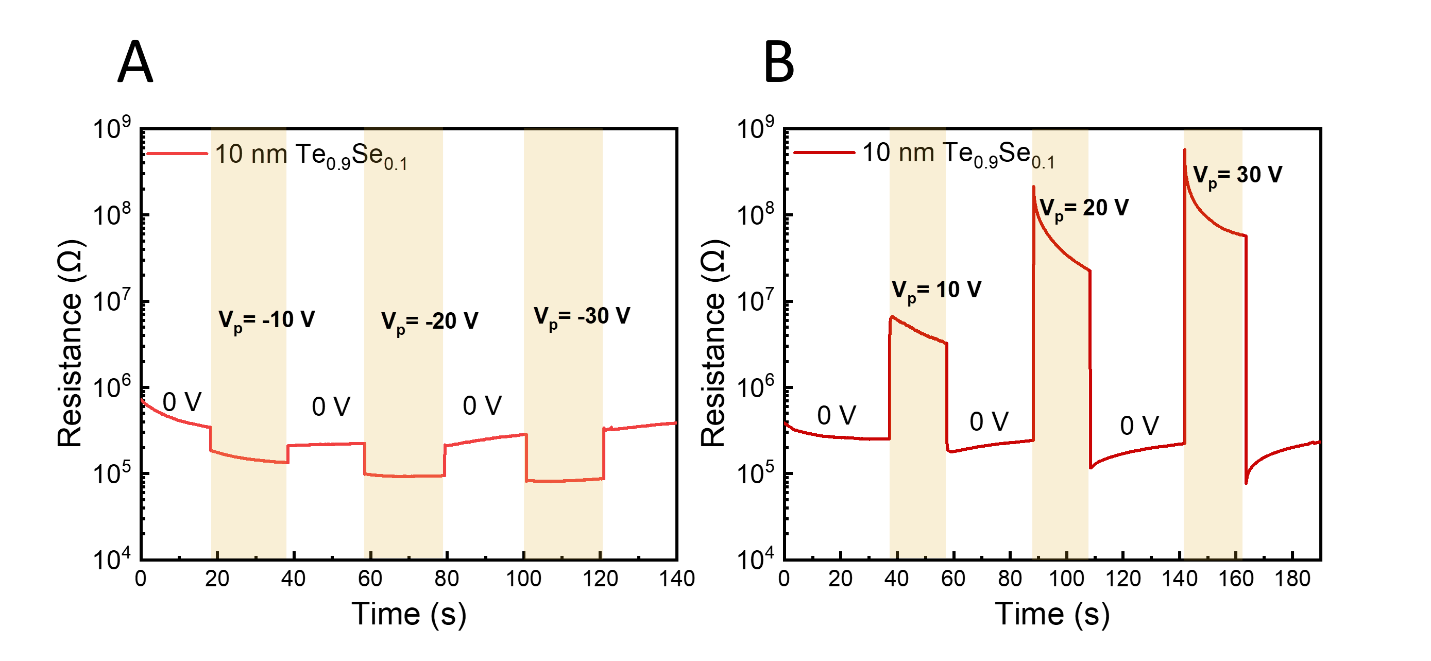


**Figure S20** **Quasi-nonvolatile switching of Te_0.9_Se_0.1_ after applying multiple gate pulse voltages.** (A) negative (V_p_), with the pulse width of 20 s. (B) positive V_p_ with a pulse width of 20 s, exhibiting multilevel resistive states.

**Supporting note 2:**

Synaptic plasticity

The behavioral model was built to capture the non-linear weight update. The conductance change with number of pulses is described by the given equation:

$G_{LTP}=B\left( 1-e^{-\frac{P}{A}} \right)+ G_{min}$ (1)

$G_{LTD}=B\left( 1-e^{-\left( \frac{P-P_{max}}{A} \right)} \right)+G_{max}$ (2)

$B=(G_{max}-G_{min})/(1-e^{-\frac{P_{max}}{A}})$ (3)

$G_{LTP}$ and $G_{LTD}$represent the conductance for long-term potentiation (LTP) and long-term depression (LTD), $G_{max}$ and $G_{min}$represent the maximum and minimum conductance value, $P_{max}$ is the maximum number of pulses required to switch the device from maximum to minimum conductance state. *A* is the parameter that controls the non-linear behavior of the weight update. *B* is a parameter that fits the function within the range of $G_{max}$, $G_{min}$ and $P_{max}$.

The experimental weight update was fitted using a MATLAB script. Firstly, the $P_{max}$was normalized to 1, then we tuned the normalized *A* for both LTP and LTD.

References

[1] D. J. Late, B. Liu, H. S. S. R. Matte, V. P. Dravid, C. N. R. Rao, *ACS Nano* 2012, **6**(6): p. 5635-p. 5641.

[2] Y. Porter, K. M. Ok, N. S. P. Bhuvanesh, P. S. Halasyamani, *Chemistry of Materials* 2001, **13**(5): p. 1910-p. 1915.
